# How to Best Protect Kidneys for Transplantation—Mechanistic Target

**DOI:** 10.3390/jcm12051787

**Published:** 2023-02-23

**Authors:** Sara Akalay, Sarah A. Hosgood

**Affiliations:** 1 Department of Development and Regeneration, Laboratory of Pediatric Nephrology, KU Leuven, 3000 Leuven, Belgium; 2 Department of Surgery, University of Cambridge, Cambridge CB2 0QQ, UK

**Keywords:** ischemia–reperfusion injury, kidney preservation, kidney reconditioning, machine perfusion

## Abstract

The increasing number of patients on the kidney transplant waiting list underlines the need to expand the donor pool and improve kidney graft utilization. By protecting kidney grafts adequately from the initial ischemic and subsequent reperfusion injury occurring during transplantation, both the number and quality of kidney grafts could be improved. The last few years have seen the emergence of many new technologies to abrogate ischemia–reperfusion (I/R) injury, including dynamic organ preservation through machine perfusion and organ reconditioning therapies. Although machine perfusion is gradually making the transition to clinical practice, reconditioning therapies have not yet progressed from the experimental setting, pointing towards a translational gap. In this review, we discuss the current knowledge on the biological processes implicated in I/R injury and explore the strategies and interventions that are being proposed to either prevent I/R injury, treat its deleterious consequences, or support the reparative response of the kidney. Prospects to improve the clinical translation of these therapies are discussed with a particular focus on the need to address multiple aspects of I/R injury to achieve robust and long-lasting protective effects on the kidney graft.

## 1. Introduction

Preserving the function of kidney grafts is imperative for effective transplantation. Static cold storage has been the gold standard in kidney transplantation for many years, and although logistically advantageous, this preservation method is unable to adequately protect kidneys from extended criteria donors (ECD) and donation after circulatory death (DCD) donors. These organs are particularly sensitive to injuries occurring during the transplantation process and lack the capacity for repair and regeneration of traditional donation after brain death (DBD) donors, putting them at risk for post-transplant graft dysfunction. These considerations often prompt the decision by the transplantation team to discard a significant portion of ECD and DCD kidneys [1,2,3,4]. Therefore, optimizing kidney preservation and promoting kidney repair will allow an increase in both the number and quality of donor kidneys. 

Typically, post-transplant graft dysfunction is the consequence of a cascade of insults occurring during the transplantation process. These insults can happen in the donor (e.g., brain death-induced inflammation, warm ischemia, drug toxicity), at the time of organ procurement (e.g., organ manipulation, warm ischemia), during the preservation and transportation phase (cold ischemia), and at the time of reperfusion. Among the various peritransplant insults, ischemia–reperfusion (I/R) injury is of particular importance as it is an inescapable phenomenon that has a profound effect on transplanted kidneys. I/R injury is not only the most important determinant of early graft function but also increases the risk of acute rejection and contributes to interstitial fibrosis and tubular atrophy, which altogether lead to long-term graft dysfunction and eventually graft loss [5]. 

I/R injury occurs as a complex interplay between pretransplant transient exposure to ischemia and subsequent reperfusion. In the past decades, considerable effort has been made to understand the causative molecular mechanisms of I/R injury and develop new therapeutic strategies. Nevertheless, despite decades of research, there is a notable contrast between preclinical successes and consistent clinical failures, which points toward a profound translational gap in the understanding of I/R injury in humans. This was recently illustrated in a systematic review comparing preclinical with clinical data on metabolic changes occurring in renal I/R injury. The authors showed poor alignment of the clinical observations with preclinical results and identified profound methodological heterogeneity in the preclinical models with regard to the diagnosis/definition of I/R injury, techniques applied to induce I/R injury, animal species and strains used, functional read-outs for kidney function/injury, sampling methods, and follow-up period [6]. Moreover, while animal models are essential in preclinical mechanistic research, a confounding impact due to interspecies differences and variations in resilience to I/R injury is inevitable and should always be considered. Another possible explanation for the poor translation of the preclinical data is systemic drug delivery; intravenous administration of any type of treatment against I/R injury makes it susceptible to removal from the circulation by the mononuclear phagocyte system, resulting in poor delivery to the appropriate organ/tissue [7]. Therefore, direct delivery to the kidney might improve treatment efficacy and reduce systemic adverse effects. Finally, both rodent and human studies to date have typically focused on treating single mechanisms of I/R injury [8]. Considering that the pathophysiology of I/R injury is multifactorial, it is likely that effective treatments will need to be capable of addressing the various deleterious aspects and immune responses involved. 

In the present review, we first discuss the biological processes implicated in I/R injury based on the currently available (pre-)clinical evidence. We then highlight the importance of ameliorating I/R injury and the potential consequences on clinically relevant outcomes. In the third section, we outline strategies to prevent or treat I/R injury from a mechanistic viewpoint. In the final section, we discuss future perspectives for kidney graft assessment and management, including considerations to improve the clinical translation of treatment strategies. 

## 2. Major Biological Processes Implicated in Ischemia–Reperfusion Injury

**Figure 1:**
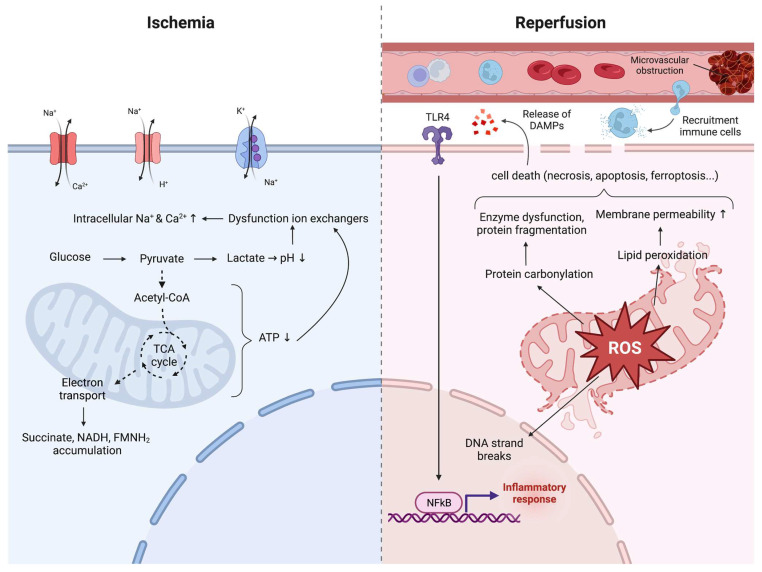
Simplified scheme of renal ischemia–reperfusion injury pathophysiology. DAMP, damage-associated molecular patterns; ROS, reactive oxygen species; TLR4, Toll-like receptor 4.

I/R injury is characterized by a restriction of blood supply to an organ followed by restoration of blood flow and reoxygenation. In the setting of kidney transplantation, this event can cause the clinical syndrome of acute kidney injury (AKI) and lead to delayed graft function (DGF), i.e., the requirement for dialysis within the first week after transplantation. Histologically, I/R injury is marked by tubular necrosis, loss of proximal tubular brush border, widespread inflammation, loss of structure, and cyst formation [9]. The pathophysiology of I/R injury is complex and involves a multitude of molecular pathways and signaling cascades. If we were to simplify this injury, we could pragmatically differentiate it into (1) the ischemic phase, (2) the reperfusion phase, and (3) the phase of immune system involvement. Successively, these events have an impact on cell and ultimately organ fate. High energy demand and strong dependence on aerobic metabolism make the tubular epithelial cells the primarily, though not solely, affected cells. Following the injury, cells can either undergo cell death or initiate a repair process that can be adaptive or maladaptive depending on the surrounding microenvironmental factors from the tubular, interstitial, and vascular compartments [10]. What follows is a summary of the current insights into the biological processes dominating each phase of I/R injury, as illustrated in Figure 1. We note that, although categorizing the injury in three different phases facilitates insights, one must keep in mind that it is an oversimplification of the various interactions between the different biological processes and the way they are interlinked with each other.

### 2.1. The Ischemic Phase

Ischemia, the disruption of blood flow, causes tissue oxygen levels to drop rapidly (hypoxia) and switches cellular metabolism from aerobic to anaerobic. Intracellular levels of adenosine triphosphate (ATP) fall precipitously, and lactate accumulation causes a decrease in cellular pH (i.e., cellular acidosis). ATP depletion and cellular acidosis both impair the normal functioning of the Na^+^/K^+^ ATPase pump along with other (ATP-dependent) ion exchangers, thereby increasing levels of intracellular sodium, calcium, and hydrogen ions. Intracellular calcium levels are further augmented by the inhibition of calcium reuptake into the endoplasmic reticulum due to ATP depletion. The osmotic imbalance caused by high intracellular sodium and calcium levels will provoke an influx of water—cellular edema—which then further disrupts cellular function as well as the structural integrity of the cell membrane and organelles. In particular, mitochondria become progressively compromised due to ATP depletion, a lack of ion homeostasis, calcium overload, and changes in pH. Upon rupture of the cell membrane, cells undergo necrosis and release endogenous molecules termed damage-associated molecular patterns (DAMP) that activate cell surface receptors called pattern-recognition receptors (PRR) on endothelial cells, tissue macrophages, and tubular epithelial cells. The activation of these PRR subsequently trigger the transcription of inflammatory genes (see Section 2.3) [11,12]. 

**Figure 2:**
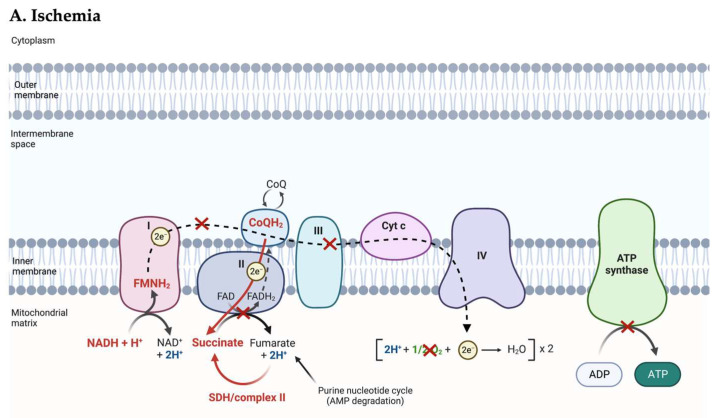

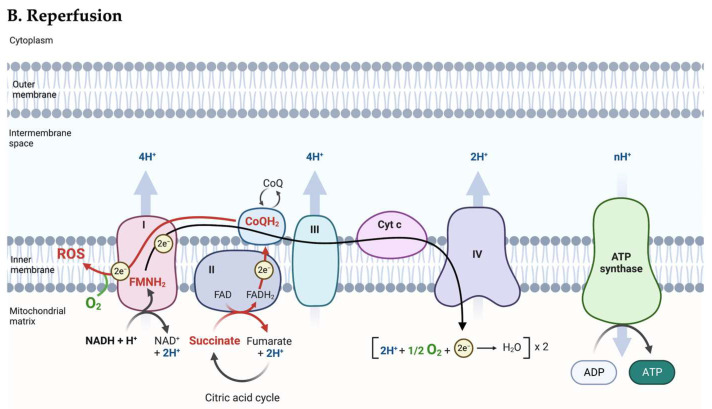
Mechanism of mitochondrial ROS production in renal ischemia–reperfusion injury. In normoxic conditions, the forward electron transport (FET) chain transfers electrons from NADH and FADH_2_ to oxygen, the final electron acceptor, through a series of redox reactions. These reactions enable the proton pumps (complex I, III, IV) to build up a H^+^ gradient which will drive the ATP synthase (complex V) to generate ATP through H^+^ backflow. During ischemia, FET is interrupted due to the absence of oxygen leading to maximal reduction of the electron donors and carriers (e.g., FMNH_2_, CoQH_2_, NADH, FADH_2_) and accumulation of succinate. During early reperfusion, the accumulated succinate is rapidly oxidized to fumarate, generating too much ubiquinol (QH_2_) which impairs FET and favors reversed electron transport (RET) to flavin mononucleotide (FMN). Reduced FMN (FMNH_2_,) is the direct source of ROS production at complex I (superoxide). CoQ, coenzyme Q or ubiquinone; Cyt C, cytochrome C; FAD, flavin adenine dinucleotide; FMN, flavin mononucleotide; NAD, nicotinamide adenine dinucleotide; SDH, succinate dehydrogenase. Black lines indicate FET; red lines indicate RET.

At the mitochondrial level, the lack of oxygen as the final electron acceptor causes an interruption of forward electron transport (FET) (Figure 2). In normoxic conditions, complex I of the mitochondrial respiratory chain is the entry point for electrons from NADH. It contains one molecule of flavin mononucleotide (FMN), which serves as the electron acceptor for NADH, and a series of iron–sulfur (Fe-S) clusters that provide a pathway for the FET to the coenzyme Q/ubiquinone (CoQ). The transfer of electrons continues down complex II, III, and IV through a series of redox reactions until electrons are passed to oxygen, the final electron acceptor, which is reduced to water. Each reaction releases energy that is converted to a proton-motive force, generating a proton gradient across the mitochondrial membrane. This proton gradient is used to catalyze the conversion of ADP to ATP by ATP synthase (complex V). In the absence of oxygen, FET is interrupted, causing the respiratory chain, redox-active enzymes, and electron carrier pools to become maximally reduced, resulting in the accumulation of NADH, succinate, FMNH_2_, and CoQH_2_ [13]. These will be the source of reactive oxygen species (ROS) generation upon reperfusion of the tissue. In other words, the ischemic period primes the tissue for subsequent ROS production by the accumulation of reduced metabolites, and the longer the ischemic period, the more pronounced this effect [14]. This is supported by the fact that I/R injury requires a sufficiently long period of ischemia in order to produce a significant amount of mitochondrial ROS upon reperfusion and that this period varies with species and tissue [15,16].

### 2.2. The Reperfusion Phase

Ischemic cells will eventually die if blood flow is not restored. Yet, it is during the reperfusion phase that most of the I/R damage is initiated. Thus, paradoxically, the essential therapeutic intervention to treat ischemia, which is reperfusion, drives tissue pathophysiology. 

#### 2.2.1. ROS Production

The first damaging event upon reperfusion is a burst of ROS production. Previously, ROS production was thought to be due to nonspecific dysregulation of the electron transport chain level, with electrons leaking at multiple nonspecific sites when oxygen was reintroduced. However, Chouchani et al. identified a conserved specific metabolic pathway in which ROS are generated by reverse electron transport (RET) at complex I of the electron transport chain (Figure 2) [15]. This process is a universal metabolic signature in a wide range of tissues (heart, liver, brain, kidney) and produces far more superoxide than any other mitochondrial process. A detailed review of the mechanism of RET and mitochondrial ROS production has been written [15]. In summary, RET occurs when electrons are forced backward through complex I from where they reduce oxygen to superoxide (^•^O_2_^−^), which is the precursor to most other ROS, including hydrogen peroxide (H_2_O_2_), hydroxyl ion (OH^−^), and hydroxyl radical (^•^OH). This reaction is driven by the accumulation of succinate during ischemia due to the reverse action of succinate dehydrogenase (SDH) or complex II, an enzyme that participates both in the citric acid cycle and the electron transport chain [14]. The accumulation of succinate is driven by the buildup of (1) NADH that transfers electrons to the CoQ pool via complex I and (2) fumarate by the degradation of AMP among other pathways [15].

Upon reperfusion, rapid consumption of accumulated succinate—within 5 min of reperfusion—generates excessive QH_2_, resulting in RET at complex I with subsequent ROS production. ROS will cause direct and extensive cellular and mitochondrial damage through lipid peroxidation, protein carbonylation, DNA strand breaks, and protein sulfhydryl oxidation, resulting in a disruption of ATP generation, enzyme dysfunction, dysregulation of calcium levels, disruption in membrane structures, and induction of the mitochondrial permeability transition pores (mPTP). mPTP cause mitochondria to become permeable to molecules smaller than 1.5 kDa, which, once inside, draw water in by increasing the organelle’s osmolar load. This event may lead mitochondria to swell and cause the outer membrane to rupture, releasing cytochrome *c.* Cytochrome *c* can in turn cause the cell to undergo apoptosis by activation of the caspase-signaling cascade. 

The wealth of evidence for succinate-driven ROS formation in I/R injury has come from studies in rodent models, and some authors have questioned the translatability of these findings to humans [14,17]. Wijermars et al. found a decline in tissue succinate content following progressive ischemia in prereperfusion biopsies of human kidneys [18]. From these data, they questioned whether succinate accumulation is a universal metabolic signature of ischemia. Considering the consistent failure of clinical studies with ROS scavenging therapy [19,20], the authors even challenged a dominant role for excess ROS formation in clinical I/R injury in kidney transplantation. Others argue that an interspecies difference in mechanisms of mitochondrial ROS generation is unlikely given the very early evolutionary origin of mitochondria [21]. Therefore, it remains essential to rule out differences in experimental methodology as a source of conflicting data.

#### 2.2.2. Postreperfusion Metabolic Paralysis

There is accumulating evidence that metabolic dysfunction has a role as a driver of I/R injury [14]. A clinical study involving living donors, deceased donors without DGF, and deceased donors with DGF showed an association between impaired postreperfusion metabolic recovery and the incidence of DGF [22]. Whereas reperfused living donor grafts and deceased donor grafts without DGF showed an instantaneous recovery of aerobic metabolism following reperfusion, DGF grafts did not. This was supported by persistent postreperfusion lactate release, a persistently low tissue glucose/lactate ratio, and prolonged release of hypoxanthine, a metabolite of ATP catabolism, in DGF grafts. Persistent anaerobic respiration and impaired functional recovery in the presence of an adequate nutrient and oxygen supply is suggestive of mitochondrial failure following reperfusion, which was confirmed by histological evaluation of mitochondria and functional measurements through respirometry following simulated I/R injury in vitro. Overall, this study suggested that preserving mitochondrial integrity and metabolic competence is critical in the prevention of DGF. In a follow-up study, the same group integrated metabolomic data from 18 pre- and postreperfusion tissue biopsies with 36 sequential arteriovenous blood samples from human kidney grafts of the three donor types [23]. They identified a clearly distinctive metabolic signature in DGF grafts characterized by impaired recovery of the high-energy phosphate-buffer phosphocreatine postreperfusion, as well as by persistent postreperfusion ATP/GTP catabolism and significant ongoing tissue damage. The impaired recovery of high-energy phosphate occurred despite the activation of glycolysis, fatty acid oxidation, and glutaminolysis and was found to be related to a defect at the level of the oxoglutarate dehydrogenase complex in the citric acid cycle. Hence, from this study, the picture emerges of DGF/clinical I/R injury being a consequence of an almost instantaneous and persistent postreperfusion metabolic failure consisting of severely impaired oxidative phosphorylation and compensatory activated normoxic glycolysis that is unable to sustain energy/ATP homeostasis. In turn, high-energy phosphate pools are progressively exhausted, and cellular integrity cannot be preserved, resulting in ongoing tissue damage. The authors concluded that efforts aimed at preventing DGF should focus on conserving metabolic competence. Remarkably, the observed metabolome of clinical DGF sharply contrasts with reported metabolic responses for rats, mice, and pigs, which all indicate reinstatement of oxidative phosphorylation within minutes of reperfusion [14,24,25,26].

#### 2.2.3. Endothelial Dysfunction and Microvascular Ischemia

Upon reperfusion, it has been demonstrated that, at the microvascular level, the renal blood flow is only partially restored with a reduction in total blood flow of up to 50% after reperfusion [27]. The absence of adequate perfusion of a previously ischemic area after reperfusion has been described as the “no-reflow” phenomenon and appears to be multifactorial with a prominent role for endothelial dysfunction [28]. Endothelial cells injured during I/R injury develop a vasoconstrictive, proinflammatory, and procoagulant phenotype [29]. On one hand, hypoxia causes the loss of intercellular contacts in injured endothelial cells, increasing vascular permeability and causing fluid loss to the interstitial space (i.e., edema) [30,31]. The interstitial edema compresses the capillaries and reduces microvascular blood flow. In addition, it has been reported that increased oxidative stress leads to an imbalance of vasodilatory and vasoconstrictive mediators in the endothelium and epithelium of the postischemic kidney, impairing microvascular autoregulation of blood flow [32]. Renal arterioles exhibit impaired vasorelaxation in response to vasodilators, such as acetylcholine (ACh), and increased responsiveness to vasoconstrictive agents, including endothelin 1, angiotensin II, thromboxane A2, and prostaglandin H2. This vasoconstriction can be enhanced by reduced nitric oxide (NO) synthesis during reperfusion due to decreased endothelial NO synthase (eNOS) expression. The vascular lumen is further narrowed due to leukocyte recruitment, accumulation, and adhesion, which also increases endothelial damage by sustaining the inflammatory and cytotoxic response. Finally, proinflammatory cytokines will activate the coagulation pathways with the subsequent formation of microthrombi and deposition of fibrin [33,34]. These obstructions impair microvascular perfusion and lead to reduced oxygen and nutrient delivery in the affected regions [35].

Because the regenerative capacity of endothelial cells is limited, microvascular damage can lead to permanent capillary rarefaction, which is a reduction in vascular density [36,37,38]. Capillary density declines rapidly within days of the initial insult and persists for weeks in parallel with an increase in peritubular matrix deposition [39]. Loss of peritubular capillaries favors chronic hypoxia, leading to overexpression of hypoxia-inducible factor 1α (HIF-1α) and favoring transcription of fibrogenic genes, such as transforming growth factor β (TGF-β) and connective tissue growth factor (CTGF). It also favors the accumulation of α-smooth muscle actin (α-SMA) positive myofibroblasts through epithelial/endothelial-to-mesenchymal transition [37]. In kidney transplant patients, peritubular capillary loss, assessed by comparing capillary density on 3-month post-transplant biopsies with capillary density on preimplantation biopsies, is strongly associated with interstitial fibrosis, tubular atrophy, and graft dysfunction 1 year post-transplant [39]. 

### 2.3. Immune System Activation

Tissue damage can also occur indirectly through the activation of the immune system. The bridge between cellular injury and innate immune system activation is the release of DAMP by necrotic cells. DAMP can be cytosolic (e.g., heat-shock proteins (HSP)), nuclear (e.g., high mobility group box 1 (HMGB1)), mitochondrial (e.g., mitochondrial ROS, mitochondrial DNA, cytochrome *c*, succinate…), or extracellular (e.g., fibronectin, hyaluronic acid, heparan sulfate) in origin and are sensed by resident kidney cells and innate immune cells through PRR present on the cell surface. PRR are families of germline-encoded proteins that can recognize DAMP and subsequently activate a downstream inflammatory response to control or repair cell damage. Activation of these receptors leads to the production of proinflammatory cytokines and chemokines, chemotaxis, opsonization, and activation of leukocytes. Over the past few decades, different PRR families have been identified, such as NOD-like receptors (NLR), Toll-like receptors (TLR), absent in melanoma-2 (AIM2)-like receptors (ALR), c-type lectin receptors (CLR), and retinoic acid-inducible gene 1-like receptors (RLR) [40,41]. Of particular relevance in renal I/R injury is the TLR family, and multiple studies suggest that ischemia upregulates TLR2 and TLR4 expression in tubular epithelial cells, as determined across various I/R injury models in vitro and in vivo [42,43,44,45]. TLR-mediated signal transduction causes activation of the transcription factor nuclear factor–κB (NF-κB), which then promotes NLRP3, pro-IL-1β, and pro-IL-18 expression. NLRP3 will assemble with pro-caspase-1 and ASC to form the NLRP3 inflammasome. Assembled NLRP3 inflammasome is then activated by ion disorders, lysosomal leakage, and mitochondrial disruption and can convert pro-caspase-1 into caspase-1. Caspase-1 then activates pro-IL-18 and pro-IL-1β to their mature forms. Mature IL-1β and IL-18 can stimulate the recruitment of innate immune cells (neutrophils, NK cells, macrophages) and the release of other cytokines. These mature cytokines are secreted into tissues to amplify inflammatory responses and cause organ damage [12]. At the same time, endothelial cells of the microvasculature upregulate adhesion molecules and have increased permeability. The net result is a sterile inflammatory process causing the already injured tissue to suffer from another hit of cytotoxic processes. Additionally, immature dendritic cells can be activated by DAMP to undergo maturation and subsequently activate the adaptive immune system in a direct manner through antigen presentation to B- and T-lymphocytes or indirectly through cytokine signaling. 

Activation of the complement system is another critical player in the innate immune response to I/R injury. The release of DAMP, intracellular antigens, and immune complex formation can activate the complement cascade by any of the three main pathways (classical, alternative, and lectin pathway) [46]. The subsequent formation of the membrane attack complex (MAC, C5b-C9) induces direct cell lysis and tubulointerstitial injury. In addition, activation of the complement cascade results in the cleavage of components C3 and C5 and the generation of anaphylatoxins (C3a and C5a), potent amplifiers of the inflammatory response causing chemotaxis, neutrophil, and macrophage activation, and the release of opsonins, such as C3b, which are important mediators in the process of antigen presentation [47,48].

### 2.4. Possible Cell Fates following I/R Injury

#### 2.4.1. Cell Death

**Figure 3:**
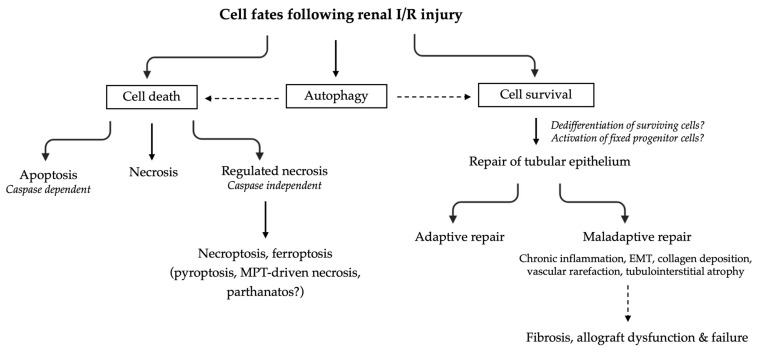
Possible cell fates following ischemia–reperfusion injury. MPT, mitochondrial permeability transition; EMT, epithelial-to-mesenchymal transition.

Tubular cell death by necrosis or apoptosis is a central feature of I/R injury (Figure 3). Necrosis is a passive process by which cells die following an overwhelming chemical or physical insult (e.g., cell edema). Cells typically exhibit cytoplasmic and mitochondrial swelling with loss of plasma membrane integrity resulting in the release of proinflammatory intracellular contents and DAMP. Hence, necrotic cell death is highly immunogenic. Apoptosis on the other hand is a tightly regulated ATP-dependent process of programmed cell death orchestrated by the caspase signaling cascade. Apoptotic cells are characterized by nuclear and cytoplasmic condensation, DNA fragmentation, cell membrane blebbing with the presence of apoptotic bodies, and externalization of phosphatidylserine, which is a signal for macrophages to engulf and remove the cells, without mounting an inflammatory response [49]. There is, however, evidence that the macrophages that engulf apoptotic cells can subsequently release HMGB1 and mitochondrial DNA (proinflammatory mediators), as well as IL-10 (anti-inflammatory mediator) [50].

More recently, new pathways in which cells die in a highly regulated manner independently of the caspase signaling and while having the morphologic features of necrosis have been described as contributing to I/R injury. These pathways are referred to as regulated necrosis (RN) and include necroptosis, ferroptosis, pyroptosis, mitochondrial permeability transition (MPT)–driven necrosis, and parthanatos (Figure 3). The common endpoint of regulated necrosis is cell membrane rupture, resulting in the release of cytosolic components and subsequent inflammation. The most extensive evidence linking RN to renal I/R injury has been gathered for necroptosis and ferroptosis and has been reviewed by Pefanis et al. [49]. In brief, necroptosis is a cell death dependent on the cytoplasmic molecules RIPK1 and RIPK3 (receptor-interacting protein kinase 3), also termed the necrosome, and their substrate MLKL (mixed lineage kinase domain-like protein). Phosphorylation of MLKL by the necrosome drives oligomerization of MLKL, allowing MLKL to insert into and permeabilize plasma membranes and organelles, leading to the expulsion of DAMP into the extracellular space. The best-studied trigger of necroptotic cell death is TNFα; however, necroptosis can also be initiated by other members of the TNFα death ligand family, such as Fas ligand and TNF-related apoptosis-inducing ligand, as well as interferons, Toll-like receptor signaling, and viral infections. Ferroptosis is another form of RN that occurs due to excessive and uncontrolled formation of lipid hydroperoxides, the product of lipid peroxidation by excess ROS formation, leading to plasma membrane rupture. During I/R injury, several processes contribute to ferroptosis: (1) the overproduction of ROS by mitochondria, NADPH oxidase, NO synthase, and xanthine oxidoreductase; (2) the reduction in glutathione and consequently glutathione peroxidase 4 activity, the sole cellular defense mechanism that can repair oxidized phospholipids, and (3) the accumulation of iron due to the degradation of intracellular ferritin, which will lead to hydroxyl radical (^•^OH) production through the Fenton reaction (Fe^2+^ + H_2_O_2_ → Fe^3+^ + ^•^OH + OH^−^). ^•^OH is highly detrimental to cell structures and can cause cardiolipin oxidation and phospholipid peroxidation. This unique lipid peroxidation signature leads to the loss of plasma membrane integrity by an unknown mechanism. This growing knowledge on understanding how cells die in renal I/R injury can eventually guide the development of novel therapeutic interventions. Nevertheless, the question remains how safe it is to inhibit nonapoptotic cell death pathways since these pathways also function as a backup system when apoptosis/caspase-signaling fails or is inhibited for instance, by caspase inhibitor expressing viruses.

#### 2.4.2. Autophagy

In certain conditions, cells can preserve their metabolic function and escape cellular death due to the lysosomal degradation of damaged cell parts (protein aggregates and organelles). This process, termed autophagy, is continuously active at low basal levels, preserving cellular homeostasis, but is stimulated upon stress through various signals like nutrient deprivation, ROS formation, or hypoxia [51,52,53]. In the context of renal I/R injury, autophagy is considered a double-edged sword that can both prevent and assist cell death, depending on the type and duration of the stress [54,55,56]. This is suggested to be dependent on the dynamics of autophagy, where excessive or uncontrolled autophagy could trigger the initiation of cell death. Decuypere et al. observed that autophagic activity depended on the severity of ischemic stress, with severe I/R injury being associated with a higher autophagic activity. Moreover, trehalose, an autophagy inducer, seemed to have a dual effect following severe ischemia: a (slight) exacerbation of renal injury 24 h post-reperfusion on the one hand and an improvement in the kidney tubular structure and reduced apoptosis 7 days following the ischemia on the other hand [57]. Based on this dual role for autophagy in renal I/R injury, the goal would be to restrict autophagy within a protective window. 

#### 2.4.3. Cellular Adaptive and Maladaptive Repair

Other than cell death, both human and animal studies have shown that tubular epithelium, especially from the proximal tubule, can undergo intrinsic repair processes after kidney injury. The cellular mechanism for that repair has been the topic of vigorous debate with two dueling methods being put forward: dedifferentiation of surviving proximal tubule cells or the existence of a fixed progenitor cell type [58,59,60,61]. On the one hand, a strong body of evidence suggests that surviving proximal tubule cells have the capacity to dedifferentiate, losing their brush border and terminal differentiation markers and acquiring a transient mesenchymal phenotype. They then re-enter the cell cycle to undergo proliferative expansion and redifferentiation, reconstituting the tubule. On the other hand, a separate body of evidence implicates the existence of a fixed intratubular proximal tubule progenitor cell selectively activated after injury. Irrespective of the source of regeneration, during this repair process, a subset of the injured proximal tubular cells can be diverted to a nonproductive senescent state, called a maladaptive or failed repair state, that is characterized by a proinflammatory and profibrotic molecular signature. Senescent tubular epithelial cells remain metabolically active and adopt a senescence-associated secretory phenotype (SASP) that releases proinflammatory cytokines and profibrotic factors contributing to interstitial fibrosis and AKI to CKD transition. Processes associated with maladaptive repair are epithelial-to-mesenchymal transition, expansion of interstitial fibroblasts and myofibroblasts, collagen deposition, vascular rarefaction, and chronic inflammation [62]. To date, mechanistic factors enabling productive, adaptive repair versus persistent, maladaptive injury states remain poorly understood and are the focus of active research. In particular, a lot of progress has been made through the application of single-cell technologies and spatial transcriptomics in mouse models of I/R injury [63,64]. For example, single-nucleus RNA sequencing identified distinct, late-stage injury, proximal tubule cell states (*Vcam1^+^*/*Ccl2^+^* with upregulation of NFκB, tumor necrosis factor, and AP-1 signaling pathways) associated with the AKI-CKD transition and SASP [65,66,67]. The current knowledge on molecular patterns of adaptive and maladaptive repair obtained from omics technologies has been reviewed by Gerhardt and McMahon. [68,69] Ultimately, if the molecular pathways regulating such important biological processes can be elucidated, targets or bioactive molecules to augment repair responses can be developed. An important challenge herein will be the translation of the findings to the repairing human kidney; until now, the bulk of the evidence is based on the evaluation of murine I/R models.

## 3. Attenuating Ischemia–Reperfusion Injury to Overcome the Shortage of Kidney Grafts

To overcome the severe shortage of kidneys for transplantation, two main strategies can be adopted at the same time: (1) improving the function and survival of currently transplanted organs and (2) enabling the use of organs that are currently rejected. As depicted below, finding a way to prevent or treat I/R injury will benefit both strategies. 

Regarding the first strategy, there is accumulating evidence that I/R injury not only is a strong determinant of early renal graft dysfunction but also reduces long-term graft survival through the reduction of renal functional mass, graft vascular injury, chronic hypoxia, and fibrosis [5,70]. Moreover, there is an observed correlation between the duration of cold and warm ischemia and the subsequently increased disturbance of adaptive immunity, immunologic recognition, and graft rejection, suggesting that I/R injury increases graft immunogenicity [71]. As rejection is the predominant cause of graft failure, one can suppose that I/R injury indirectly increases the risk of graft loss through increased immunogenicity and thereby creating a higher risk of allograft rejection. 

Regarding the second strategy, many organs are currently not transplanted because factors such as age or background chronic pathology make them unlikely to recover substantially from I/R injury and be able to sustain adequate kidney function for years after transplantation [72]. These are typically organs retrieved from ECD donors, which are DBD donors over the age of 60 or between 50 to 59 years old with comorbidities (history of hypertension, terminal serum creatinine >1.5 mg/dl, or death from a cerebrovascular accident). Moreover, an increasing proportion of organs are now retrieved from DCD donors where the organs not only undergo a cold ischemic injury but are also exposed to a period of warm ischemia occurring in the donor. This additional period of warm ischemia significantly increases the ischemic injury to the organs and doubles the likelihood of DGF after kidney transplantation, explaining the greater reluctance to accept DCD kidneys for transplantation [73,74,75]. UK Transplant Registry data indicate that around 13% of DCD donor kidneys are currently discarded after retrieval; in the US, discard rates as high as 20% have been reported [76,77]. DCD donors can be further categorized as controlled DCD (cDCD), where organs are retrieved following circulatory arrest after the planned withdrawal of life-sustaining therapy in a hospital, or uncontrolled DCD (uDCD), where organs are retrieved from patients who have an unexpected cardiac arrest without return of spontaneous hemodynamic activity following advanced life support. Consequently, the warm ischemic time is much longer for uDCD than for cDCD grafts. Although several European countries, such as Spain, France, and The Netherlands, have used uDCD donors to expand the donor pool, other countries, such as the UK and the United States, have made minimal use of these donor kidneys [77,78,79,80]. A single-center cohort study from Spain with a follow-up period of 10 years indeed showed higher rates of primary nonfunction (PNF) and DGF in uDCD; PNF incidence was three times higher in uDCD (12.3%) compared to DBD (4.1%), and DGF incidence was almost four times higher in uDCD (65.1%) compared to DBD (17.7%) [79]. 

It is important to mention that while DCD kidneys have a higher incidence of PNF and DGF than DBD kidneys, the long-term graft survival of functioning DCD kidneys is equivalent to that in recipients of DBD kidneys. This observation is supported by large registry analyses from the UK, the US, and the Netherlands [77,80,81]. Furthermore, regarding the graft function, there is no evidence that 5-year graft function is inferior in recipients of kidneys from DCD donors. Although there are insufficient data to comment reliably on graft function in the much longer term, it seems very unlikely, given the 5-year data, that it will be inferior to that of DBD donor kidneys. It, therefore, seems that DCD kidneys—both controlled and uncontrolled—could contribute even more to the donor pool if the PNF and DGF rate was somehow improved. Notably, these observations also suggest a differential impact of DGF on DBD and DCD kidneys. Indeed, Brook et al. compared the outcome between DCD and DBD grafts that developed DGF and found that graft survival up to 6 years after transplantation was better for DCD than DBD kidneys [82]. One possible explanation for this phenomenon is that the development of DGF in DBD grafts requires a more severe insult. This hypothesis was evaluated in 6635 kidney transplants performed in the Netherlands [83]. In this cohort, shorter dialysis periods and a superior post-transplant estimated glomerular filtration rate (eGFR) were seen in DBD grafts compared to DCD grafts, showing that the differential impact was not caused by a more severe DGF phenotype in DBD grafts. Therefore, the authors hypothesized that the differential impact reflects differences in graft resilience to I/R injury, with DCD donor kidneys being more resilient than DBD grafts. It is currently not clear whether this assumption stands and which mechanisms would underly this increased resilience, although it may be related to the brain death-induced inflammatory storm [84]. 

In conclusion, preventing or treating I/R injury will likely benefit all kidney grafts from deceased donors irrespective of the type of donor but, in particular, uDCD kidneys, as this donor pool remains a largely unutilized resource. Moreover, as the incidence of DGF is directly proportional to the ischemic time, strategies to prevent or treat I/R injury might also allow a safe extension of preservation time without compromising transplant outcomes [85]. 

## 4. Strategies to Protect the Kidney Graft

**Figure 4:**
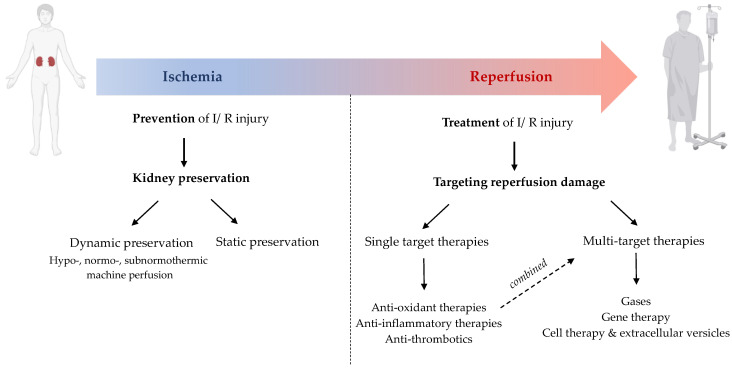
Strategies for kidney graft protection during transplantation.

Often, post-transplant graft dysfunction (i.e., DGF) is considered to be the consequence of the reperfusion injury due to the various deleterious processes described above (oxidative stress, endothelial dysfunction, immune system activation…). Nevertheless, recipients of living donors experience only rarely graft dysfunction despite the kidney graft being exposed to reperfusion. This indicates the important contribution of the preceding ischemic insult in the manifestation of clinical I/R injury. Therefore, prevention of I/R injury would consist of abrogating (the processes of) ischemia, which is achieved through improving kidney preservation. Treatment of I/R injury involves therapies that target the consequences of reperfusion injury (Figure 4). 

### 4.1. Improving Kidney Preservation

Even prior to the first successful kidney transplantation in 1954, scientists understood the necessity of optimal organ preservation, i.e., putting an organ into a state that allows later reactivation and restitution of its original function with the aim of minimizing damage during the period of mal- and nonperfusion [86]. Reviewing the history of organ preservation, we note that overall, the field has progressed from dynamic to static modalities and from normothermic to hypothermic preservation back and forth. In the early days, organs were perfused with blood at physiological temperatures. Later, scientists found that the use of lower temperatures can attenuate organ damage during perfusion by abating cellular metabolism. Thereafter, it became standard practice to statically preserve organs at hypothermic temperatures. In recent years, with the demand to expand the organ donor pool, the status of organ preservation saw a retrospective shift from static cold storage (SCS) to earlier techniques of dynamic preservation, as these techniques provide great potential for improved graft preservation, viability assessment, and repair/regeneration [87].

#### 4.1.1. Hypothermic Machine Perfusion (0–8 °C)

As stated above, the concept of hypothermic machine perfusion (HMP) is not new. Early research in this field dates back to the 1960s when Belzer designed a clinical machine perfusion device, leading to the first successful human HMP kidney transplant in 1968. Dynamic preservation was the only way to preserve deceased organs until cheap SCS solutions were introduced by Collins in the 1970s and 1980s [88]. SCS offered a simple and effective way to preserve and transport organs and became the dominant method of kidney preservation. However, since the expansion of DCD programs, HMP is now considered the standard method of kidney preservation in the majority of European countries for DCD kidneys. Studies have shown that HMP can reduce the risk of DGF and PNF and improve 1-year graft survival compared to SCS [89]. The exact working mechanism of HMP is incompletely understood and presumed to be multifactorial. Similar to SCS, during HMP, the rate of metabolism is reduced to ∼10% compared to physiological temperature. Although this does not prevent cold-related depletion of ATP and accumulation of metabolic products in the graft, the dynamic manner of preservation gives beneficial effects [90]. The continuous or pulsatile flow of the perfusion solution results in a continual flush of the microcirculation and decreases the accumulation of debris and toxic metabolites. Furthermore, the flow generates mechanical vasodilatation and vascular shear stress, enhancing eNOS phosphorylation, NO-dependent vasodilatation, and molecular vasoprotection (decreased epithelial-to-mesenchymal transition, vimentin, fibrosis, endothelin, innate immunity, TLR4, HMGB1, cytokines, and increased protective endothelial genes, HIF-1α and nitric oxide signaling) [91,92,93].

Although the clinical benefits of using HMP have been demonstrated, it is important to mention that HMP in itself does not have any conditioning effect on the kidney graft, i.e., it does not improve kidney quality but rather slows down the detrimental effects occurring during the cold ischemic time and helps maintain kidney quality, as it was at the time of retrieval. This implies that during HMP, graft function still depends on the duration of the cold ischemic time, and as such, HMP does not allow a safe extension of the preservation time before transplantation. Similar to cold storage, every additional hour of cold storage time will increase the risk of graft failure after transplantation [94].

The addition of oxygen during HMP can support a low level of ATP synthesis. This may reduce the accumulation of succinate and protect against I/R injury, as shown in experimental models [13]. In clinical practice, a randomized controlled trial led by Jochmans et al. comparing oxygenated and nonoxygenated HMP in DCD kidneys from donors over 50 years found no significant improvement of oxygenated HMP on graft function at 1 year [95]. However, incidences of biopsy-proven rejection and postoperative complications were significantly lower. Interestingly, in a rodent model of hypothermic oxygenated perfusion (HOPE), allograft treatment with HOPE not only protected against preservation injury but also impressively downregulated the immune system, blunting the alloimmune response [96]. Therefore, oxygenation under hypothermic conditions may have some favorable effect on immune activation and warrants further investigation.

#### 4.1.2. Normothermic Machine Perfusion (35–38 °C) 

The term normothermia usually refers to the physiological body temperature of the species used in the study, which is 37 °C for human and rodent studies and 38 °C in studies with porcine models. The idea underlying the technique is to replicate the normal metabolism of the kidney outside the body, providing oxygen and essential substrates in an environment maintained at a physiological temperature. Normothermic perfusion techniques are gradually being reintroduced in solid organ transplantation and offer many advantages when compared to hypothermic preservation [97,98,99]. Firstly, aerobic metabolism can be restored, which minimizes or even avoids the cold ischemic insult. Secondly, the kidney can be maintained in a state allowing close observation and assessment of viability. Lastly, it provides the opportunity to add therapies to a functioning organ to directly manipulate and improve its condition. The safety and feasibility of normothermic machine perfusion (NMP) in kidney transplantation have been reported in several clinical studies [100,101,102]. To date, NMP has been used to resuscitate the kidney for a short duration (typically 1 h) at the recipient center after a period of hypothermic preservation. NMP shows promise in improving early graft function and is capable of assessing quality [103]. Nonetheless, further application is needed to determine the true value of NMP in clinical practice. 

Extending the duration of NMP to reduce the effects of cold ischemic injury has proven to be beneficial in the experimental setting [104,105,106]. Consequently, with advances in technology, there is a growing interest to strive for ischemia-free kidney transplantation [107]. A case report demonstrated the use of the Kidney Assist device to perfuse a kidney from a DBD donor from the time of retrieval until transplantation [108]. Although logistically and technically difficult, the authors demonstrated proof of principle that it is possible to avoid ischemic injury using this approach [107]. This strategy will, however, not be applicable to all donors (i.e., uDCD donors) and faces several challenges, such as the technical complexity, the requirement for a portable kidney NMP device, the possibility of long-distance transportation, and an evaluation of the cost-effectiveness. 

#### 4.1.3. Subnormothermic Machine Perfusion (20–34 °C)

Subnormothermic machine perfusion (SNMP) is a midway approach between HMP and NMP. It was proposed to achieve a balance between the cytoprotective effects of reduced cellular metabolism under hypothermic temperatures while having sufficient metabolism to allow viability assessment and potential organ repair and reconditioning. In addition, grafts can be perfused at subnormothermic temperatures without the need for an oxygen carrier, simplifying the procedure, reducing cost, and preventing the harmful effects of hemolysis and free heme [109,110,111,112]. Although initial studies have shown that kidneys perfused with SNMP are superior to grafts preserved under cold storage, a more recent study showed that porcine kidneys preserved under SNMP were associated with higher indices of renal and tubular injury upon reperfusion than those preserved under NMP [113,114,115]. Clearly, more evidence is required on SNMP. 

#### 4.1.4. Controlled Oxygenated Rewarming

Following cold ischemic preservation, the abrupt change in temperature from hypothermia to normothermia may trigger mitochondrial dysfunction as well as mitochondrial proapoptotic signal transduction [116,117]. Controlled oxygenated rewarming (COR) is an alternative organ perfusion method in which the perfusion begins at hypothermic temperatures and gradually increases to subnormothermia, improving restitution of cellular homeostasis and mitigating rewarming injury by an adapted increase in temperature and metabolism. Minor and colleagues demonstrated that COR following SCS had better kidney function with mitigated activity of mPTP opening, caspase 9 activation, and apoptosis in porcine kidneys when compared to hypothermic preservation [118]. It is presumed that the controlled transition from hypo- to subnormothermia protects cold-preserved mitochondria from initial dysfunction and that the brief subnormothermic perfusion period helps to reverse mitochondrial dyshomeostasis prior to the challenge of normothermia. 

### 4.2. Targeting Kidney Repair and Regeneration

For lower-quality kidneys, specifically ECD and DCD kidneys, improving preservation will not be sufficient to guarantee optimal graft function. Additional measures that can either target the detrimental biological processes of I/R injury and/or support the repair/regenerative response of the kidney will likely result in improved utilization of these kidneys. 

To treat renal I/R injury, many interventions have been trialed in both rodent and clinical studies with the majority of these studies focusing on treating single mechanisms of the injury. Unfortunately, none of these interventions have reached clinical practice so far. As stated before, based on the intricate nature of I/R injury, it is likely that effective therapeutic treatments will need to be capable of addressing the different biological aspects and immune responses involved. This “multitarget” strategy can be achieved either by combining agents that each work on different pathways or by utilizing a therapy that can modulate several protecting/deleterious pathways. Moreover, difficulties remain with systemic delivery where toxicity or insufficient targeting of the damaged organ limits the therapeutic potential of many of the trialed agents. Theoretically, any drug that is typically delivered systemically can also be introduced during machine perfusion for more concentrated treatments; for this reason, we expect machine perfusion to facilitate the application and translation of treatments for I/R injury. In recent years, the feasibility of administering pharmacologic agents as well as cellular, genetic, or biological therapies directly to the kidney graft during dynamic preservation has been demonstrated, supporting the complementarity between machine perfusion and these therapies [119]. What follows is an overview of these agents classified according to their protective mechanism (Table 1). 

#### 4.2.1. Therapies Targeting a Single Biological Process of I/R Injury

##### Targeting Oxidative Stress

Conventional antioxidants (e.g., glutathione, vitamin E, ubiquinol) have been unsatisfactory in preventing mitochondrial oxidative damage in large part due to their low penetration to the mitochondrial interior [120]. The inner mitochondrial membrane is highly impermeable and rich in cardiolipin and maintains a strong negative internal potential that is required for the function of the electron transport chain. For this reason, mitochondria-targeting antioxidants were developed to improve the delivery to the mitochondrial interior. To date, different mitochondria-targeting antioxidants have been studied, the most prominent of which are MitoQ and SS-31 [121]. 

MitoQ is a chimeric molecule consisting of the lipophilic cation triphenylphosphonium (TPP) conjugated with an antioxidant moiety, the ubiquinone that is also found in coenzyme Q10. The negatively charged mitochondrial membrane potential and the positive charge of the lipophilic cation drive the transport of this cationic antioxidant into mitochondria, resulting in a concentration 10,000 times higher in the mitochondrial matrix than in the cytosol. By the action of complex II in the mitochondrial respiratory chain, the ubiquinone part of MitoQ is rapidly activated to the active ubiquinol antioxidant. After detoxifying ROS, the ubiquinol part of MitoQ is converted to ubiquinone, which is again subjected to complex II to be recycled back to active antioxidant ubiquinol [122]. In a recent study, Hamed et al. demonstrated stable and concentration-dependent uptake of MitoQ in pig and human kidneys using either SCS, HMP, or NMP [123]. Total blood flow and urine output were significantly greater in pig kidneys treated with 50 mmol/l MitoQ compared with controls. In human kidneys, although not statistically significant, a trend towards increased blood flow, urine output, and creatinine clearance was observed in kidneys treated with MitoQ compared to controls. This improvement in human kidney function was associated with the downregulation of inflammatory pathways, including TNFα signaling via NF-κB and IL15RA, consistent with the anti-inflammatory effects of MitoQ reported in previous studies [124,125,126].

Szeto-Schiller peptide SS-31 (also known as MTP-131, elamipretide, or Bendavia) is a short tetrapeptide that selectively concentrates in the inner mitochondrial membrane where it binds to and stabilizes cardiolipin and prevents its peroxidation [127]. SS peptides feature a common structural motif of alternating aromatic (Phe, Tyr, and Dmt (2′,6′- dimethyltyrosine)) and basic (Arg, Lys) residues and freely penetrate membranes in a potential-independent manner due to their aromatic-cationic amino acid sequence. Tyr or Dmt residues are responsible for the ROS scavenging abilities of these peptides. Besides scavenging ROS, SS-31 also inhibits mPTP and thereby prevents the mitochondrial release of cytochrome *c* and subsequent apoptosis [128]. In a rat model of renal I/R injury, treatment with SS-31 protected mitochondrial structure and respiration during early reperfusion, accelerated recovery of ATP, reduced apoptosis and necrosis of tubular cells and abrogated tubular dysfunction [129]. In a phase 2a prospective multicenter randomized placebo-controlled study Saad et al. assessed the safety, tolerability, and efficacy of intravenously administered elamipretide (clinical formulation of SS-31) for reduction of reperfusion injury in patients with severe atherosclerotic renal artery stenosis undergoing revascularization with percutaneous transluminal renal angioplasty (PTRA) [130]. Patients were treated before and during PTRA with elamipretide (0.05 mg/kg per hour intravenous infusion) or placebo. Compared to the placebo group, the patients who received elamipretide showed increased eGFR and a decline in systolic blood pressure after 3 months. 

Besides the aforementioned antioxidant agents, there is a growing interest in ferroptosis inhibitors to attenuate I/R injury. As stated earlier, ferroptosis is a form of programmed, iron-dependent cell death driven by the depletion of cellular antioxidant defenses such as glutathione and overwhelming lethal lipid peroxidation. Hence, iron chelators (e.g., Dexrazoxane, Deferoxamine), agents increasing the expression of glutathione peroxidase 4 (GPX4) but also specific ferroptosis inhibitors such as Ferrostatin-1 and Liproxstatin, can be potentially therapeutic in renal I/R injury [131]. Liproxstatin-1 was reported to be able to suppress ferroptosis in human renal proximal tubule epithelial cells, in GPX4^−^/^−^ kidneys, and in I/R-induced tissue injury models [132]. Currently, the understanding of the role of ferroptosis in I/R injury and acute kidney injury is still in its infancy, and the current research progress has been reviewed elsewhere [49,133]. 

##### Targeting Microvascular Ischemia

A study conducted in 17 living donor kidneys and 28 deceased donor kidneys compared microthrombi and fibrin deposition in glomeruli and peritubular capillaries in preimplantation and postreperfusion biopsies [134]. In the preimplantation biopsies, a higher number of microthrombi were observed in deceased donor kidneys compared to living donor kidneys. This difference disappeared after reperfusion with an equal number of microthrombi/mm^2^ in both groups, indicating that the majority of microthrombi and fibrin depositions are not donor-derived but develop upon reperfusion of the graft. In addition, it appeared that kidneys transplanted in recipients who were administrated 5000 IU of unfractionated heparin before clamping of the vessels showed significantly fewer microthrombi upon reperfusion than kidneys transplanted in recipients without heparin. Hence, the administration of anticoagulants and/or thrombolytics at the most crucial moment of microthrombi formation (i.e., reperfusion) could improve microcirculation and potentially even graft outcome.

Di Rito et al. showed that prolonged cold storage promotes fibrinogen production and accumulation within the tubular epithelium [135]. Upon restoration of physiological temperature during NMP or graft implantation, rapid fibrinogen secretion to urine and the microvasculature in combination with red blood cell aggregation causes microvascular obstructions occurring within 15 min after normothermic temperature restoration and impairing adequate graft perfusion. The investigators subsequently delivered tissue plasminogen activator (tPA) with plasminogen at the beginning of 1 h of NMP of nontransplantable human kidneys and managed to clear microvascular obstructions completely. This resulted in more stable perfusion parameters, decreased levels of NGAL, IL-6, and ICAM-1, and increased urine production [119].

Prior to this study in NMP, other groups attempted ex vivo thrombolytic treatment of human kidneys during HMP. Nghiem et al. showed a reduction in glomerular thrombosis from 50% to 23% after 12–16 h of HMP with tPA in DCD kidneys with extensive glomerular thrombosis on procurement biopsies [136]. Improvement in the biopsy appearance allowed successful transplantation of these kidneys that otherwise would have been discarded based on biopsy findings. In a prospective randomized controlled trial of tPA during HMP of 12 kidney pairs, no significant difference in DGF or death-censored graft survival could be observed. While there was a trend toward lower creatinine and higher glomerular filtration rates in the tPA group at 3, 6, 9, and 12 months, these differences were not significant [137]. It should be considered that this trial was limited in sample size, that tPA activity is reduced at lower temperatures, and that the dose of tPA used was lower than the dose used by Nghiem et al. Moreover, considering that most of the microthrombi develop after reperfusion, coating grafts with anticoagulants, such as heparin, during machine perfusion to prevent thrombi formation could be a more potent alternative. Thrombalexin, a synthetic thrombin inhibitor, and Corline heparine conjugate (CHC), a macromolecular heparin conjugate, have both been tested during HMP of porcine kidney grafts followed by ex vivo reperfusion [138,139]. The results suggested improved perfusion and improved organ function in the early period after reperfusion. A study with porcine DCD kidneys perfused with the combination of Lys-plasminogen, antithrombin-III, and tPA showed that the treated kidneys had reduced vascular resistance and provided good function when transplanted [140]. Very recently, the safety and tolerability of CHC therapy during HMP of human kidneys before transplantation was demonstrated in a randomized phase 1 clinical trial [141]. Overall, these results encourage further investigations of local anticoagulant and/or thrombolytic therapy during kidney transplantation.

##### Targeting Inflammatory Pathways and the Immune System

A recent systematic review by De Beule et al. found that during machine perfusion, perfusate cytokine levels are similar in kidneys perfused with whole blood compared to kidneys perfused with leukocyte-depleted packed red blood cells [142]. This demonstrates that kidney tubular epithelial cells and the resident immune cells can produce large amounts of cytokines and chemokines, which can upregulate inflammatory pathways within the graft before implantation and independently of the presence of an immune system. Proinflammatory mediators will aggravate the severity of I/R injury through the recruitment of leukocytes upon transplantation and the removal of secreted cytokines and chemokines from the kidney during machine perfusion could be a strategy of organ preparation before implantation. This question was analyzed in two experimental studies using the Cytosorb^®^ haemadsorber, a filter that effectively reduces a range of cytokines up to a size of 60 kDa. Hosgood et al. incorporated the Cytosorb^®^ filter into the perfusion circuit during NMP of porcine kidneys after 22 h of cold ischemia. Cytosorb^®^ significantly reduced the levels of proinflammatory cytokines IL-6 and IL-8 and improved renal blood flow but had no influence on renal function as measured by urine output and creatinine clearance [143]. Possibly, this can be related to the fact that due to its broad-spectrum action, the Cytosorb^®^ adsorber also removes potentially important anti-inflammatory mediators. In a follow-up study, the same group perfused nontransplantable human kidneys with a haemadsorber and studied the effect on the graft transcriptome [144]. They first showed that NMP itself induces the expression of oxidative phosphorylation pathway genes and inflammatory pathways genes (e.g., TNFα signaling via NFκB), supporting the idea that during NMP inflammatory mediators are released from the kidney into the perfusion circuit, recirculate and stimulate proinflammatory gene transcription. Notably, there was a significant positive correlation between the upregulation of inflammatory genes and the duration of DGF, with a greater enrichment of these pathways in kidneys that experienced more prolonged DGF after transplantation. Conversely, the length of DGF negatively correlated with the magnitude of expression of oxidative phosphorylation pathway genes, which has the potential to restore ATP synthesis and cellular homeostasis. Therefore, the authors were able to identify a DGF-associated gene signature where higher expression of TNFα signaling via NFκB and lower expression of oxidative phosphorylation genes was associated with longer DGF. The addition of a haemadsorber to the perfusion circuit significantly reduced inflammatory gene expression but also increased oxidative phosphorylation and fatty acid metabolism pathways. These changes in gene expression support the idea that Cytosorb^®^ can be clinically beneficial. Nevertheless, more experimental and clinical studies are warranted, and it is worth noting that although Cytosorb^®^ is already used in clinical practice in a variety of systemic hyperinflammatory conditions, robust data from randomized controlled trials showing a beneficial effect of Cytosorb^®^ in any of these conditions is lacking [145]. 

**Table 1:** Therapies for kidney ischemia–reperfusion (I/R) injury including examples of available studies. ETC, electron transport chain; NA, not available; ROS, reactive oxygen species.

Target	Class	Drug or Intervention	Mechanism of Action	Studies in Kidney I/R Injury Model	
				Ex Vivo	In Vivo
**Oxidative stress**	Mitochondria-targeting antioxidants	MitoQ (quinone)	Accumulation at the matrix-facing surface of the mitochondrial membrane where complex II of the ETC reduces it into the active ubiquinol form (MitoQH2). Ubiquinol directly scavenges mitochondrial ROS.	[123]	[17,146]
		SS-31 peptide	Largely unknown; interacts with cardiolipin in the inner mitochondrial membrane; ROS scavenging activity	NA	[129,130]
	Ferroptosis inhibitors	See “cell death”			
	Nanozymes	Cerium oxide nanozymes, noble metal nanozymes (Pt, Au, Ag…), etc.	Nanomaterials exhibiting the properties of some natural antioxidant enzymes, such as superoxide dismutase, glutathione peroxidase, and catalase	NA	[147,148]
**Microvascular ischemia**	Fibrinolytics	tPA	Serine protease catalyzes the conversion of (fibrin-bound) plasminogen to plasmin; plasmin causes the degradation of fibrin clots	[135,136,140]	NA
	Anticoagulants	Corline heparine conjugate (CHC), Thrombalexin	Prevention of thrombus formation	[138,139,140]	[149,150,151]
**Inflammation & immune system**	Cytokine filter	Cytosorb^®^	Removal of circulating cytokines up to 60 kDa	[143,144]	NA
	TNFα inhibitor	Etanercept	Blocking of TNFα inflammatory cascade; blocking of TNFα also indirectly prevents necroptosis	[152]	[153]
	Immune system	Machine perfusion	Elimination of kidney resident immune cells	[154]	NA
	Complement system	C1 esterase inhibitor, eculizumab, mirococept	Inhibition of the complement cascade	[155]	[156,157,158,159,160]
**Cell death**	Ferroptosis inhibitors	Iron chelators: Deferoxamine, Dexrazoxane	Reduction of intracellular iron, thereby preventing ROS production through the Fenton reaction and iron-dependent initiation of phospholipid peroxidation	[161]	[162,163,164]
		Inhibitors of lipid peroxidation: Liproxstatin, Ferrostatin-1, Quercetin, 16–86	Elimination of lipid hydroperoxides	NA	[132,165,166]
		GPX4 inducers: Galangin, Carvacrol	Detoxification of lipid peroxides through GPX4	NA	[167]
	Necroptosis inhibitors	Necrostatin-1, Sorafenib, necrosulfonamide	Inhibition of RIPK1 and/or RIPK3 (Necrostatin-1 and Sorfanib); inhibition of MLKL (necrosulfonamide)	NA	[168,169,170,171]
**Multitarget**	Cell therapy	MSC, MAPC, nKSPC	Antioxidant, anti-inflammatory, restoration of metabolism, autophagic regulation, antiapoptosis (see Table 2)	[172,173,174,175,176,177,178]	[179]
	Gene therapy	siRNA, miRNA	Silencing of one or multiple deleterious genes at the posttranscriptional level (e.g., caspase-3, C3)	[180]	[181]
	Gases	CO, NO, H_2_S	Anti-inflammatory, antiapoptotic, antithrombotic, and vasodilatory properties; ROS scavenging (H_2_S)	[182,183,184,185,186,187]	[188]
**Other**	Biological therapies	Etanercept, CD47 blocking antibody	CD47 limits renal recovery, cell survival, and self-renewal after I/R injury; Etanercept: see “inflammation and immune system”	[189,190,191,192]	[153]

An alternative strategy to haemadsorption is the targeting of one individual proinflammatory cytokine that plays a central role in the pathogenesis of I/R injury. Diuwe et al. hypothesized that the administration of the TNFα inhibitor Etanercept could improve outcomes following kidney transplantation. Randomization of 94 DBD kidneys to HMP with or without Etanercept did not demonstrate significant differences concerning kidney machine perfusion parameters nor did the authors observe significant changes regarding DGF, rejection episodes, or allograft survival [152]. As for all studies evaluating pharmacological interventions in HMP, it must be considered that the efficacy of the tested agent might improve when administered during NMP in a fully metabolically active organ. Unexpectedly, the proportional hazard Cox model showed that Etanercept caused a 2.3-fold increase in the risk of recipient’s death and a 2.6-fold increased risk of graft loss. 

Rather than modulating cytokines, some groups have focused on the role of kidney resident immune cells during machine perfusion. In past years, it has become increasingly clear that nonlymphoid tissues, including the kidney, are populated by a resident immune system [193]. The kidney not only hosts tissue-resident macrophages but also harbors lymphocytes, including innate lymphoid cells, natural killer (NK) cells, natural killer T cells, mucosal-associated invariant T cells, and γδ T cells [86,194]. With the current gold-standard cold storage preservation, the organs are transplanted with their resident immune cells into the recipients in whom direct allorecognition between donor-derived antigen-presenting cells and recipient T cells can trigger an immune response [195]. The Manchester group analyzed donor-derived leukocytes during 6 h of NMP of porcine kidneys and demonstrated a rapid immune cell efflux from the donor kidney into the NMP circuit, including major populations of T cells as well as cells with antigen-presenting capacity, such as classical and nonclassical monocytes, macrophages, and B cells [154]. NK cells, mature and immature neutrophils, eosinophils, and basophils were also detected. The authors suggested that the migration of billions of donor leukocytes in conjunction with the secretion of inflammatory cytokines would drive direct allorecognition and would orchestrate significant recipient immune activation following homing of donor leukocytes to recipient lymph nodes after transplantation. Therefore, they proposed that NMP could be used to clear organs of resident immune cells and remove the inflammatory burden of donor kidneys before transplanting them. This hypothesis will need further exploration, especially since it was demonstrated that it is the exosomes released from donor organ parenchymal cells rather than the passenger leukocytes that initiate alloreactive T cell responses after transplantation [196]. 

Another area of interest in decreasing the inflammatory burden of I/R injury is the inhibition of the complement system. Experimental studies using complement-deficient rodent models have confirmed the beneficial effects of counteracting complement activation on the prevention or mitigation of I/R injury [197,198]. Consequently, many interventions on the level of C1, C3, C5, and convertases are being evaluated in preclinical and clinical studies. In two studies performed in a nonhuman primate model of kidney transplantation, both donor and recipient systemic treatment with C1 esterase inhibitor reduced the incidence of DGF [156,157]. A phase 1/2 randomized controlled trial investigating the safety and efficacy of C1 esterase inhibitor among deceased donor kidney transplant recipients at a high risk for DGF showed a shorter duration of DGF and lower incidence of graft failure among C1 esterase inhibitor-treated patients [158,159]. Eculizumab (Soliris^®^), an anti-C5 monoclonal antibody, has been investigated for the prevention of DGF in two randomized controlled trials. The safety profile was good, but eculizumab administration prior to reperfusion had no effect on the incidence of DGF [160]. The EMPIRIKAL trial was the first clinical study to investigate the ex vivo delivery of a complement inhibitor (Mirococept) in deceased donors. Mirococept, a C3 convertase inhibitor, contains a membrane-targeting tag allowing for retention of the therapeutic moiety in the donor kidney after treatment. As the initially calculated dose of 10 mg failed to show a significant difference from the control in the first cohort of patients; the study was stopped to conduct a dose-finding study in normothermic machine-perfused porcine kidneys [155]. The porcine kidney study identified an optimal dose of 80 mg (equivalent to 120 mg in a human kidney) that provided a basis for further clinical development.

#### 4.2.2. Multitarget Therapies

##### Cellular Therapies and Cell-Derived Extracellular Vesicles

Over the past decade, the use of cell-based therapies in solid organ transplantation has been proposed as an innovative approach to induce immune tolerance, attenuate organ damage caused by I/R injury, and potentiate regeneration. Although these cell-based treatments are inherently more complex to develop than classical drugs and come with additional logistical and regulatory challenges, they have the capacity to act on a multitude of biological processes and therefore could be transformative for the transplantation field. In the context of ex vivo kidney perfusion, three different cell sources have been studied thus far: mesenchymal stromal cells, multipotent adult progenitor cells, and, very recently, neonatal kidney stem/progenitor cells (Table 2).

Mesenchymal stromal cells (MSC) are a population of adult, multipotent stromal cells of mesodermal origin with in vitro abilities of self-renewal and differentiation potential into three mesenchymal lineages (i.e., adipocytes, chondrocytes, osteocytes). They can be isolated from diverse sources such as bone marrow, adipose tissue, and the umbilical cord, and their low immunogenicity make them amenable to allogeneic “off the shelf” use [199]. Their proposed therapeutic effect is attributed to their anti-inflammatory and immunosuppressive properties as well as the promotion of tissue repair through the release of paracrine mediators (cytokines, growth factors, extracellular vesicles) from their secretome. Brasile et al. were the first to perfuse human kidneys with MSC using their Exsanguinous Metabolic Support (EMS) platform. They first conducted a dose-finding study showing that at a dose of 2 × 10^8^, MSC perfusion parameters were adversely affected. They therefore selected a dose of 1 × 10^8^ MSC and perfused 5 kidney pairs at subnormothermic temperature (32 °C) for 24 h. MSC treatment led to a significant increase in ATP synthesis, growth factors associated with regenerative pathways after ischemic insults, and mitosis along with normalization of metabolism and the cytoskeleton [178]. Regarding the fate of the infused MSC, more than 95% of the MSC were recovered from the recirculated perfusate, providing evidence that the MSC did not integrate into the renal tissue but rather remained in circulation. 

Later, the international MePEP consortium (MEsenchymal stem cell administration and normothermic machine PErfusion in Porcine renal transplantation) was set up to evaluate MSC therapy during ex vivo perfusion of porcine kidneys. In the first study, different doses (0, 10^5^, 10^6^, or 10^7^) of human MSC were evaluated during 7 h of NMP of porcine kidneys [173]. Intact MSC were visualized inside the lumen of glomerular capillaries but only when infused numbers were as high as 10^7^. MSC did not appear to migrate into the kidney parenchyma. Moreover, the authors demonstrated a decrease in circulating MSC during the perfusion. This was the case in perfusions with and without a kidney in the perfusion circuit, which is probably the result of cells being exposed to pressure and flow during the perfusion itself. In a follow-up study, they evaluated the effect of bone marrow- and adipose tissue-derived human MSC on kidney function. They were unable to discern specific differences between the two MSC sources and neither MSC treatment improved renal function nor the histological evaluation [172]. However, the injury markers NGAL, NAG, and LDH were significantly lower in MSC-treated kidneys while there was a significant increase in IL-6 and IL-8, both important proinflammatory markers. Thereafter, the consortium evaluated MSC treatment in NMP using a porcine renal autrotransplantation model. The kidneys underwent 14 h of HOPE followed by 4 h of NMP with an infusion of 10^7^ human or porcine adipose-derived MSC. All kidneys were autotransplanted after contralateral nephrectomy. After 14 days of follow-up, no beneficial effects on kidney function, kidney injury markers, or kidney histology were observed in this early post-transplant period [175].

Another cell type evaluated for kidney graft reconditioning is the multipotent adult progenitor cells (MAPC). MAPC are adult, bone marrow-derived stromal cells that are inherently very similar to MSC. Both cell types can be isolated from bone marrow but are subjected to different expansion and culture protocols, resulting in phenotypically and functionally different cell types. Nevertheless, they maintain a very similar mechanism of action [200]. Thompson et al. perfused 5 pairs of discarded human kidneys randomized to receive 50 × 10^6^ MAPC during 7 h of NMP [177]. The MAPC-treated kidneys demonstrated improved urine output, decreased expression of the kidney injury marker NGAL, and improved microvascular perfusion on contrast-enhanced ultrasound. This reconditioning effect seemed to be mediated through changes in circulating cytokines and immune mediators towards an anti-inflammatory profile (decreased IL-1β, increased IL-10, increased indoleamine-2,3-dioxygenase (IDO) activity) [177]. Tracking of the MAPC revealed their localization within the glomerular capillaries and peritubular capillaries throughout the kidney with some evidence of cells crossing the vascular endothelium to reside in the perivascular space. 

Although MSC and MAPC have been the focus of most studies so far, both cell sources are not kidney-derived and lack the capacity to differentiate into functional kidney epithelial cells, which might represent an important shortcoming for kidney-targeted cell therapy [201]. Indeed, there is increasing evidence that cells keep an epigenetic memory of their tissue of origin, which may influence their potency, including the capacity for differentiation and engraftment, in disease modeling and treatment. Therefore, using kidney-derived (stem) cells is of particular interest. In 2016, Arcolino et al. described the noninvasive isolation of kidney stem-/progenitor cells from the urine of preterm neonates, termed neonatal kidney stem/progenitor cells (nKSPC) [202]. nKSPC are derived from developing kidneys, the only period in which authentic stem cells are present in the human kidney, and express SIX2, a transcription factor active in kidney cap mesenchyme cells critical for maintaining the progenitor state [203,204]. Their high proliferative capacity (> 20 passages) and their potential to differentiate into functional podocytes and proximal tubular epithelial cells make them an attractive source for kidney regenerative applications. Very recently, the group investigated the administration of nKSPC during 6 h of NMP and showed that nKSPC induced the de novo expression of SIX2 in proximal tubular epithelial cells of the donor kidneys [176]. This phenomenon was followed by the upregulation of regenerative markers such as SOX9, HIF-1α, and VEGF, suggesting that nKSPC therapy can trigger an endogenous regenerative process. Moreover, nKSPC administration significantly lowered levels of kidney injury biomarkers and reduced inflammatory cytokine levels via the tryptophan-IDO-kynurenine pathway. Tracking of the nKSPC revealed single cells/fragments in the cortex at 2 h and 4 h of perfusion, while at 6 h of perfusion, a high number of cells/fragments was observed in the medullary region. 

Although there is a growing number of experimental studies investigating the administration of cells during machine perfusion, the actual clinical relevance is not understood yet, and many important questions remain to be answered, including the optimal cell source, the exact mechanism of action, and the optimal timing, dosing, and frequency of administration. The fate and persistence of administered cells during machine perfusion and after transplantation are also incompletely understood. Once the kidney is transplanted, the cells could be rapidly removed, similar to what happens after systemic administration of MSC. Studies demonstrated a low persistence of MSC upon intravenous administration due to trapping in the microcapillary network of the lungs where most of the cells die within 24–48 h and are cleared by local macrophages [205,206,207]. Nevertheless, there is increasing evidence that MSC apoptosis and the released secretome are actually required for their therapeutic function [208,209]. Thus, apoptosis of administered cells should not necessarily be seen as disadvantageous. Finally, the optimal duration and conditions of machine perfusion also need to be established, and, if allogeneic cells are used, the consequences of allorecognition after transplantation need to be thoroughly understood.

As it is becoming increasingly clear that the therapeutic effects of MSC and cell therapies, in general, are mediated by paracrine factors—and not by differentiation to specialized cells in vivo—there is also growing interest to use extracellular vesicles (EV) as an alternative to cell therapy. EV are important mediators in paracrine intercellular communication and are secreted by cells into the extracellular space to transfer biological material, such as coding and noncoding RNA and growth factors, to modify the recipient cells. Evidence from preclinical models of kidney injury suggests that EV, particularly those released by stem cells, have therapeutic properties and can accelerate kidney recovery in models of AKI, chronic kidney disease, and kidney transplantation [210,211]. Moreover, EV may be less immunogenic, less toxic, and able to cross biological barriers more easily than their cellular counterparts. They also mitigate the risks related to the administration of viable cells, such as uncontrolled cell replication, differentiation, or vascular occlusion. To date, only a few studies have investigated the feasibility and efficacy of this strategy for kidney graft reconditioning. The delivery of MSC extracellular vesicles to rat deceased donor kidneys during 4 h of cold perfusion protected against ischemic injury [212]. Rampino et al. evaluated the administration of MSC-derived EV during HOPE of ECD human kidneys. Compared to kidneys without EV treatment, the treated kidneys had improved histology, higher levels of the growth factors VEGF and HGF, and lower caspase-3 expression [213]. During normothermic reperfusion, EV-treated kidneys had lower lactate release and higher glucose levels, suggesting that the gluconeogenesis system in the HOPE with EV group was preserved.

**Table 2:** Types of cell therapy for kidney graft reconditioning during machine perfusion. aMSC, adipose tissue-derived MSC; bmMSC, bone marrow-derived MSC; CIT, cold ischemic time; EV, extracellular vesicles; HMP, hypothermic machine perfusion; MHC, major histocompatibility complex; NMP, normothermic machine perfusion; WIT, warm ischemic time.

Cell Type	Source	Isolation	Passages in Culture	Differentiation to Functional Kidney Epithelial Cells	Mechanism of Action	Immunogenicity	Studies in Machine Perfusion			
							Study	Model	Treatment and Dose	Perfusion
**Mesenchymal stromal cells (MSC)**	Bone marrow, adipose tissue, perinatal tissues (cord blood, placenta, umbilical cord), synovial fluid…	(Semi) invasive	~max 8–12 passages	No	Paracrine effects; proangiogenic, antiapoptotic, antioxidant, anti-inflammatory and antifibrotic	Low immunogenicity (low expression of MHC class I, no expression of MHC class II and costimulatory molecules)	Gregorini et al. (2017) [212]	Rat kidneys	5 × 10^6^ MSC or EV derived from 5 × 10^6^ MSC	4 h of HMP after 20 min of WIT
							Brasile et al. (2019) [178]	Human kidneys	10^8^ MSC	24 h of SNMP after 21–43 h of CIT
							Pool et al. (2019) [173]	Porcine kidneys	10^5^ human aMSC 10^6^ human aMSC 10^7^ human aMSC Fluorescent labeled bmMSC	7 h of NMP after 30 min of WI and 3.5–5 h of SCS
							Pool et al. (2020) [172]	Porcine kidneys	10^7^ human aMSC 10^7^ human bmMSC	7 h NMP after 2–3 h of HMP and 20 min WIT
							Lohmann et al. (2021) [175]	Porcine kidneys	10^7^ porcine aMSC 10^7^ human aMSC	240 min NMP after 14 h oxygenated HMP and 75 min WIT
**Multipotent adult progenitor cells (MAPC)**	Bone marrow	Invasive	> 20 passages	No	Paracrine effects; anti-inflammatory, immunomodulatory, protolerogenic	Low immunogenicity (lower levels of MHC class I compared to MSC, no expression of MHC class II and co-stimulatory molecules)	Thompson et al. (2021) [177]	Human kidneys	5 × 10^7^ MAPC	7 h of NMP after 13–36 h of CIT
**Neonatal kidney stem-/progenitor cells (nKSPC)**	Urine	Noninvasive	> 21 passages	Yes	Reactivation of nephrogenic transcription factor SIX2, upregulation of SOX9 and VEGF; anti-inflammatory, immunomodulatory effects	Not reported	Arcolino et al. (2022) [176]	Human kidneys	10^7^ nKSPC	6 h of NMP after 17–28 h of CIT

##### Gases

Some gases, when delivered at controlled doses, have shown evidence of having protective effects on the kidney. The beneficial effects of the gasotransmitters carbon monoxide (CO), NO, and hydrogen sulfide (H_2_S) have been well-documented in small animal models and thereafter during ex vivo porcine kidney perfusion [183,214]. CO, H_2_S and, to a lesser extent, NO showed renoprotective effects throughout early functional outcome measures (renal blood flow, urine output, creatinine clearance, and kidney injury markers) [184,186,187]. Mechanistically, these gases have a wide range of protective functions, including anti-inflammatory, antiapoptotic, antithrombotic, and vasodilatory properties [215,216,217]. H_2_S also functions as an antioxidant therapy through the scavenging of ROS, increasing production of intracellular glutathione and upregulation of the antioxidant defense system through the Nrf2 pathway [218]. Unfortunately, studies in human kidneys or (auto)transplantation models are lacking and should be the focus of future research in that field. Moreover, the current development and establishment of NMP systems are of particular benefit for graft treatment with gases due to more efficient gas release at physiological temperature as well as easier control of their administration via perfusion parameters.

##### Gene Therapies

Gene therapy involves the intentional transfer of genetic material into host cells to alter their original state and thereby treat the disease. Broadly, it includes DNA-based therapeutics, which can be delivered as plasmids or integrated into a viral vector, and RNA-based therapeutics. RNA-based therapy comprises, on the one hand, mRNA-based protein replacement to be used to achieve a specific protein expression and, on the other hand, antisense oligonucleotide (ASO) and RNA interference (RNAi) (i.e., small interfering RNA (siRNA) and microRNA (miRNA)) to promote degradation of endogenous RNA transcripts and inhibit their translation [219]. These can be delivered as naked RNA or using a viral or nonviral vehicle (lipids, peptides, polymers) [220]. For in vivo clinical application, RNA therapy requires a delivery vehicle to protect the RNA from rapid degradation and achieve efficient delivery to the cells/tissue/organ of interest. These delivery vehicles are, however, not ideal in immunosuppressed transplant recipients due to concerns regarding the oncogenic potential of viral vectors and the proinflammatory immune responses seen with the nonviral vehicles [180]. Therefore, the possibility of targeted delivery to an isolated kidney using (normothermic) machine perfusion enables the use of naked RNA, decreasing the risk of potential side effects but also reducing the manufacturing cost of RNA therapy. 

For the purpose of attenuating I/R injury, the focus has been on RNA-based therapies due to their transient effect, and to date, both siRNA and miRNA have been studied during machine perfusion. siRNA and miRNA share many similarities. Both are short double-stranded RNA molecules that exert gene silencing effects at the post-transcriptional level by targeting mRNA, yet the major difference is that siRNA is highly specific with only one mRNA target, whereas miRNA has multiple targets [221]. One microRNA can inhibit more than 100 genes. Yang et al. infused naked synthetic caspase-3 siRNA directly into the renal arteries of ischemic porcine kidneys prior to 24 h of SCS followed by 3 h NMP to assess kidney function. Caspase-3 siRNA downregulated the expression of caspase-3 precursor and its active subunit, reduced significantly the levels of apoptosis, and improved kidney oxygenation and acid-base homeostasis [222]. In a follow-up study, the effect was not evident after autotransplantation, and this was possibly due to the instability of the siRNA [223]. Zheng et al. used a cocktail of siRNA to knock down C3, Fas, and RelB using a murine model. The cocktail was administered directly into the kidney via the renal artery followed by 4h of perfusion at 4 °C. Silencing of these genes during perfusion improved renal function, prolonged graft survival, and reduced inflammation and apoptosis after transplantation [224]. More recently, Thompson et al. delivered an antagomir oligonucleotide silencing miRNA-24-3p to human kidneys during NMP [180]. They first identified miRNA that were highly expressed throughout NMP and relevant in I/R injury and subsequently decided to silence miRNA-24-3p, which has detrimental effects in I/R injury. After 2 h of NMP, there was evidence of antagomir uptake by vascular endothelial cells and tubular epithelial cells. Notably, when the antagomir was delivered during HMP, it could be found coating the vascular endothelium without intracellular uptake in proximal tubular epithelial cells, suggesting that uptake is an active process that only occurs in normothermic conditions. Two target genes of miRNA-24-3p, heme oxygenase-1 (HMOX1) and sphingosine-1-phosphate receptor-1 (S1PR1), were significantly upregulated in the treated kidneys. No significant differences in NMP parameters or inflammation were noted in the treated kidneys. Although the authors were able to show successful delivery of a naked oligonucleotide to human kidneys using NMP, substantial preclinical work needs to follow to show efficacy on clinically relevant endpoints. The ability of one miRNA to affect multiple mRNA targets may be an advantage but may also be a disadvantage if protective genes are downregulated. Nevertheless, the therapeutic possibilities in this field are enormous; many miRNA could be targeted and a combination of treatments is possible.

#### 4.2.3. Other Therapies

##### Nanoparticles

Nanoparticles (NP) are drug-delivery vehicles that have been developed to overcome the limitations of free therapeutics and navigate biological barriers [225,226]. A key benefit of NP is their modularity: The composition can be adjusted depending on the type of therapeutics (small molecule, protein, nucleic acid), the desired rate of drug release, and the target cell type [227]. The three broad classes of NP are lipid-based (e.g., liposomes, lipid NP, emulsion), polymeric (e.g., dendrimer, polymer micelle, nanosphere), and inorganic NP (e.g., gold NP, silica NP, iron oxide NP), with each class having advantages and disadvantages regarding cargo, delivery, and patient response [228]. Since their introduction in the clinics in the 1990s, they have proven to be safe for use in humans in a wide variety of diseases. Nevertheless, clinical translation remains a challenge, largely due to issues with controlling NP localization following systemic administration. Once present in the blood circulation, NP are opsonized by serum proteins and subsequently eliminated by the reticuloendothelial system. Therefore, NP typically cannot efficiently escape from the vasculature to reach extravascular targets. In addition, surface adsorption of serum proteins can create a so-called “proteincorona” that can mask targeting ligands and abolish targeting benefits in vivo. Delivery of NP directly to the graft in leukocyte-depleted, serum-free perfusate during perfusion could potentially overcome these limitations. Tietjen et al. delivered proof of concept that NP can be administered during NMP and are taken up by endothelial cells. These can serve as depots for long-term drug release. Moreover, conjugation of the NP with an anti-CD31 antibody improved the accumulation of the NP in the endothelial cells by about 5- to 10-fold when compared to nontargeted NP [35].

NP can be used for encapsulation of (hydrophobic) drugs and, as mentioned previously, RNA therapy. Moreover, nanozymes, which are inorganic nanoparticles with intrinsic enzyme-like characteristics, have been booming over the past decade. In 2007, it was reported that Fe_3_O_4_ NP has intrinsic peroxidase-mimicking activity, and since that time, hundreds of nanomaterials have been found to mimic the catalytic activity of peroxidase, catalase, glutathione peroxidase, and superoxide dismutase [229,230]. Compared with traditional antioxidants, nanozyme antioxidants possess remarkable broad-spectrum ROS scavenging activity, high stability, and rapid clearance, with low price and large-scale production. Recently, Feng et al. developed gold–platinum NP with a broad ROS scavenging capacity capable of alleviating kidney I/R injury both in vitro and in vivo without obvious toxicity by suppressing cell apoptosis, inflammatory cytokine release, and inflammasome formation [148]. The NP also had an effect on inhibiting the transition to chronic kidney disease by reducing kidney fibrosis in the long term. 

##### Biological Therapies

Biological therapies include monoclonal antibodies and fusion proteins, medicines whose active drug substance is made by living organisms. Aside from the TNFα inhibitor Etanercept, the CD47-blocking antibody is another biological therapy that has been used to treat I/R injury. This cell surface molecule negatively modulates cell and tissue responses to stress through the limitation of specific homeostatic pathways and initiation of cell death pathways [231]. The effect of blocking CD47 has proven to be effective in several animal models of transplant-mediated renal I/R injury [189,190,191]. 

## 5. Challenges and Future Considerations

Much progress has been made in the field of kidney preservation, and at present, machine perfusion is making the transition into clinical practice. Simultaneously, numerous strategies to recondition kidney grafts are being investigated in the experimental setting, and for most of these treatments, applying them ex vivo during machine perfusion will improve their efficacy and eliminate many limitations of systemic in vivo delivery. These efforts and investments made by many research groups have the ultimate goal to abrogate I/R injury, transplant more kidneys of better quality, and, if possible, allow a safe extension of the preservation time. This latter could avoid overnight transplantation and hasty decision-making and offer more time for organ evaluation and allocation. Still, a lot of work has yet to be done. Regarding kidney machine perfusion, many questions need further evaluation, such as which type of perfusion modality should be applied (hypothermic, subnormothermic, normothermic), when during the transplantation process kidney perfusion should be started, how long the perfusion should last, and how we can further optimize perfusate constituents. It is conceivable that all perfusion modalities, or a combination thereof, could have a role in the clinical setting, depending on donor and organ type and the aim of the perfusion (i.e., organ preservation, quality assessment, and/or reconditioning). With regard to reconditioning therapies, there is promising experimental data, but none of these therapies has been adopted into current clinical practice. One of the translational difficulties is the complexity of I/R injury. Many pathological mechanisms contribute to I/R injury, and the probability of finding a single therapeutical agent is low. The rational combination of two or more different protection strategies or a single intervention strategy that protects against multiple targets may help produce a solid and robust renoprotective effect. Moreover, our understanding of the pathophysiology of I/R injury in human kidney grafts, if and how the donor type affects these processes, and how the kidney physiology functions during ex vivo machine perfusion is far from complete. We believe that the current implementation of multiomics research (genomics, transcriptomics, metabolomics, and proteomics) holds great potential to improve our insights on these matters. From single-cell and spatial transcriptomics we have already acquired a better understanding of adaptive and maladaptive repair after acute kidney injury [63].

Another common denominator of failed translation of preclinical data is that human kidneys, especially the ones that will be eligible for reconditioning therapies, have a certain degree of background (histological) damage and comorbidities that are usually not present in the animal testing stage. These comorbidities can include hyperglycemia, hypertension, hyperlipidemia but also aging, which are usually not present in the young, healthy animals used for research. Another variable that is often overlooked is the sex of the studied model. Most studies are performed in male animals, while there is consistent evidence of a difference between males and females regarding the time of ischemia to induce a similar extent of injury [232,233]. To reduce all these possible confounders, the establishment of preclinical animal models needs to be closer to clinical practice, and/or therapies should be evaluated in human kidneys discarded for transplantation. Another issue in preclinical research is the lack of standardization and reproducibility. As outlined in the introduction, there is profound methodological heterogeneity in animal models used for I/R injury, and the same is also true for studies evaluating reconditioning therapies in machine perfusion or in vivo. No two studies are the same with many differences in machine perfusion protocol and/or therapeutics (doses) being evaluated. Moreover, there is very little evidence that studies are repeated by independent research groups to confirm safety and efficacy. 

It is expected that, ultimately, the transplantation field will move from all-round cold storage preservation toward a kidney preservation technique with/without additional reconditioning adjusted to the needs of a specific organ. In that scenario, personalized organ-tailored formulations will be chosen after a quality assessment evaluating the degree of damage in the graft. Hence, it is necessary that the identification of reliable biomarkers (and/or imaging techniques) to assess the kidney graft quality—including the severity of kidney injury, the remaining functional capacity, and the capacity to regenerate—progresses alongside the search for reconditioning therapies. These quality criteria should be able to reliably predict post-transplant outcome in a short time window and are also indispensable to evaluate the effect of reconditioning therapies and subsequently make a decision about the “transplantability” of an organ. To date, no validated sets of on-pump viability markers exist. 

## 6. Conclusions

In this review, we detailed the current knowledge on I/R injury and highlighted the importance of adequately protecting kidneys from I/R injury in order to expand the pool of transplantable organs and reduce the number of patients on the transplant waiting list. The rapid advances made in machine perfusion techniques and the broad spectrum of reconditioning therapies being explored provide encouraging prospects. Nevertheless, further extensive experimental evidence is necessary to transfer these techniques into clinical practice. This needs to include a profound understanding of I/R injury occurring during kidney transplantation and reasonable preclinical models to evaluate therapeutic strategies. Thus far, the best strategy to protect kidneys for transplantation has not been defined but is expected to be a combination of adequate preservation and pharmacological agents or interventions acting together to exert synergistic and long-lasting protective effects for the kidney graft.

## Data Availability

Data sharing not applicable.

## Abbreviations

Ach	acetylcholine
AKI	acute kidney injury
ALR	absent in melanoma-2 (AIM2)-like receptors
α-SMA	α-smooth muscle actin
ASO	antisense oligonucleotide
ATP	adenosine triphosphate
cDCD	controlled donation after circulatory death
CHC	Corline heparine conjugate
CIT	cold ischemic time
CKD	chronic kidney disease
CLR	c-type lectin receptors
CO	carbon monoxide
CoQ	coenzyme Q, ubiquinone
COR	controlled oxygenated rewarming
CTGF	connective tissue growth factor
Cyt C	cytochrome C
DAMP	damage-associated molecular patterns
DBD	donation after brain death
DCD	donation after circulatory death
DGF	delayed graft function
ECD	extended criteria donors
eGFR	estimated glomerular filtration rate
EMS	exsanguinous metabolic support
EMT	epithelial-to-mesenchymal transition
eNOS	endothelial nitric oxide synthase
EV	extracellular vesicles
FAD	flavin adenine dinucleotide
FET	forward electron transport
FMN	flavin mononucleotide
GPX4	glutathione peroxidase 4
HIF-1α	hypoxia inducible factor 1α
HMGB1	high mobility group box 1
HMOX1	heme oxygenase 1
HMP	hypothermic machine perfusion
HOPE	hypothermic oxygenated perfusion
H_2_S	hydrogen sulfide
HSP	heat-shock proteins
IDO	indoleamine 2,3-dioxygenase
I/R	ischemia reperfusion
MAPC	multipotent adult progenitor cells
MHC	major histocompatibility complex
miRNA	microRNA
MLKL	mixed lineage kinase domain-like protein
mPTP	mitochondrial permeability transition pores
MSC	mesenchymal stromal cells
NAD	nicotinamide adenine dinucleotide
NAG	N-acetyl-β-(D)-glucosaminidase
NF-κB	nuclear factor–κB
NGAL	neutrophil gelatinase-associated lipocalin
nKSPC	neonatal kidney stem/progenitor cells
NLR	NOD-like receptors
NMP	normothermic machine perfusion
NO	nitric oxide
NP	nanoparticles
PNF	primary nonfunction
PRR	pattern-recognition receptors
PTRA	percutaneous transluminal renal angioplasty
RET	reverse electron transport
RIPK	receptor-interacting protein kinase
RLR	retinoic acid inducible gene 1-like receptors
RN	regulated necrosis
RNAi	RNA interference
ROS	reactive oxygen species
SASP	senescence-associated secretory phenotype
SCS	static cold storage
SDH	succinate dehydrogenase
S1PR1	sphingosine-1-phosphate receptor 1
siRNA	small interfering RNA
SNMP	subnormothermic machine perfusion
TGF-β	transforming growth factor β
TLR4	Toll-like receptor 4
tPA	tissue plasminogen activator
TPP	triphenylphosphonium
uDCD	uncontrolled donation after circulatory death
WIT	warm ischemic time

## References

[1] Cooper M., Formica R., Friedewald J., Hirose R., O’Connor K., Mohan S., Schold J., Axelrod D., Pastan S. (2019). Report of National Kidney Foundation Consensus Conference to Decrease Kidney Discards. Clin. Transplant..

[2] Callaghan C.J., Harper S.J., Saeb-Parsy K., Hudson A., Gibbs P., Watson C.J., Praseedom R.K., Butler A.J., Pettigrew G.J., Bradley J.A. (2014). The discard of deceased donor kidneys in the UK. Clin. Transplant..

[3] Stewart D.E., Garcia V.C., Rosendale J.D., Klassen D.K., Carrico B.J. (2017). Diagnosing the Decades-Long Rise in the Deceased Donor Kidney Discard Rate in the United States. Transplantation.

[4] Mohan S., Chiles M.C., Patzer R.E., Pastan S.O., Husain S.A., Carpenter D.J., Dube G.K., Crew R.J., Ratner L.E., Cohen D.J. (2018). Factors leading to the discard of deceased donor kidneys in the United States. Kidney Int..

[5] Zhao H., Alam A., Soo A.P., George A.J.T., Ma D. (2018). Ischemia-Reperfusion Injury Reduces Long Term Renal Graft Survival: Mechanism and Beyond. EBioMedicine.

[6] Lerink L.J.S., de Kok M.J.C., Mulvey J.F., Le Dévédec S.E., Markovski A.A., Wüst R.C.I., Alwayn I.P.J., Ploeg R.J., Schaapherder A.F.M., Bakker J.A. (2022). Preclinical models versus clinical renal ischemia reperfusion injury: A systematic review based on metabolic signatures. Am. J. Transplant..

[7] Hume D.A. (2006). The mononuclear phagocyte system. Curr. Opin. Immunol..

[8] Franzin R., Stasi A., Fiorentino M., Simone S., Oberbauer R., Castellano G., Gesualdo L. (2021). Renal Delivery of Pharmacologic Agents During Machine Perfusion to Prevent Ischaemia-Reperfusion Injury: From Murine Model to Clinical Trials. Front. Immunol..

[9] Le Clef N., Verhulst A., D’Haese P.C., Vervaet B.A. (2016). Unilateral Renal Ischemia-Reperfusion as a Robust Model for Acute to Chronic Kidney Injury in Mice. PLoS ONE.

[10] Basile D.P., Bonventre J.V., Mehta R., Nangaku M., Unwin R., Rosner M.H., Kellum J.A., Ronco C. (2016). Progression after AKI: Understanding Maladaptive Repair Processes to Predict and Identify Therapeutic Treatments. J. Am. Soc. Nephrol..

[11] Smith S.F., Hosgood S.A., Nicholson M.L. (2019). Ischemia-reperfusion injury in renal transplantation: 3 key signaling pathways in tubular epithelial cells. Kidney Int..

[12] Su X., Liu B., Wang S., Wang Y., Zhang Z., Zhou H., Li F. (2022). NLRP3 inflammasome: A potential therapeutic target to minimize renal ischemia/reperfusion injury during transplantation. Transpl. Immunol..

[13] Darius T., Vergauwen M., Smith T., Gerin I., Joris V., Mueller M., Aydin S., Muller X., Schlegel A., Nath J. (2020). Brief O(2) uploading during continuous hypothermic machine perfusion is simple yet effective oxygenation method to improve initial kidney function in a porcine autotransplant model. Am. J. Transplant..

[14] Chouchani E.T., Pell V.R., Gaude E., Aksentijević D., Sundier S.Y., Robb E.L., Logan A., Nadtochiy S.M., Ord E.N.J., Smith A.C. (2014). Ischaemic accumulation of succinate controls reperfusion injury through mitochondrial ROS. Nature.

[15] Chouchani E.T., Pell V.R., James A.M., Work L.M., Saeb-Parsy K., Frezza C., Krieg T., Murphy M.P. (2016). A Unifying Mechanism for Mitochondrial Superoxide Production during Ischemia-Reperfusion Injury. Cell Metab..

[16] Parekh D.J., Weinberg J.M., Ercole B., Torkko K.C., Hilton W., Bennett M., Devarajan P., Venkatachalam M.A. (2013). Tolerance of the human kidney to isolated controlled ischemia. J. Am. Soc. Nephrol..

[17] Dare A.J., Bolton E.A., Pettigrew G.J., Bradley J.A., Saeb-Parsy K., Murphy M.P. (2015). Protection against renal ischemia-reperfusion injury in vivo by the mitochondria targeted antioxidant MitoQ. Redox Biol..

[18] Wijermars L.G., Schaapherder A.F., Kostidis S., Wüst R.C., Lindeman J.H. (2016). Succinate Accumulation and Ischemia-Reperfusion Injury: Of Mice but Not Men, a Study in Renal Ischemia-Reperfusion. Am. J. Transplant..

[19] Firuzi O., Miri R., Tavakkoli M., Saso L. (2011). Antioxidant therapy: Current status and future prospects. Curr. Med. Chem..

[20] Suzuki K. (2009). Anti-oxidants for therapeutic use: Why are only a few drugs in clinical use?. Adv. Drug Deliv. Rev..

[21] Martin J.L., Gruszczyk A.V., Beach T.E., Murphy M.P., Saeb-Parsy K. (2019). Mitochondrial mechanisms and therapeutics in ischaemia reperfusion injury. Pediatr. Nephrol..

[22] Wijermars L.G.M., Schaapherder A.F., de Vries D.K., Verschuren L., Wüst R.C.I., Kostidis S., Mayboroda O.A., Prins F., Ringers J., Bierau J. (2016). Defective postreperfusion metabolic recovery directly associates with incident delayed graft function. Kidney Int..

[23] Lindeman J.H., Wijermars L.G., Kostidis S., Mayboroda O.A., Harms A.C., Hankemeier T., Bierau J., Sai Sankar Gupta K.B., Giera M., Reinders M.E. (2020). Results of an explorative clinical evaluation suggest immediate and persistent post-reperfusion metabolic paralysis drives kidney ischemia reperfusion injury. Kidney Int..

[24] Wei Q., Xiao X., Fogle P., Dong Z. (2014). Changes in Metabolic Profiles during Acute Kidney Injury and Recovery following Ischemia/Reperfusion. PLoS ONE.

[25] Fonouni H., Esmaeilzadeh M., Jarahian P., Rad M.T., Golriz M., Faridar A., Hafezi M., Jafarieh S., Kashfi A., Yazdi S.H.F. (2011). Early Detection of Metabolic Changes Using Microdialysis During and After Experimental Kidney Transplantation in a Porcine Model. Surg. Innov..

[26] Keller A.K., Jorgensen T.M., Ravlo K., Nielsen T.K., Olsen L.H., Stolle L.B. (2009). Microdialysis for Detection of Renal Ischemia After Experimental Renal Transplantation. J. Urol..

[27] Molitoris B.A., Sutton T.A. (2004). Endothelial injury and dysfunction: Role in the extension phase of acute renal failure. Kidney Int..

[28] Rezkalla S.H., Kloner R.A. (2002). No-reflow phenomenon. Circulation.

[29] Molitoris B.A. (2014). Therapeutic translation in acute kidney injury: The epithelial/endothelial axis. J. Clin. Investig..

[30] Ogawa S., Gerlach H., Esposito C., Pasagian-Macaulay A., Brett J., Stern D. (1990). Hypoxia modulates the barrier and coagulant function of cultured bovine endothelium. Increased monolayer permeability and induction of procoagulant properties. J. Clin. Investig..

[31] Ogawa S., Koga S., Kuwabara K., Brett J., Morrow B., Morris S.A., Bilezikian J.P., Silverstein S.C., Stern D. (1992). Hypoxia-induced increased permeability of endothelial monolayers occurs through lowering of cellular cAMP levels. Am. J. Physiol..

[32] Legrand M., Mik E.G., Johannes T., Payen D., Ince C. (2008). Renal hypoxia and dysoxia after reperfusion of the ischemic kidney. Mol. Med..

[33] Hoffmann J.N., Vollmar B., Laschke M.W., Fertmann J.M., Jauch K.W., Menger M.D. (2005). Microcirculatory alterations in ischemia-reperfusion injury and sepsis: Effects of activated protein C and thrombin inhibition. Crit. Care.

[34] Sharfuddin A.A., Sandoval R.M., Berg D.T., McDougal G.E., Campos S.B., Phillips C.L., Jones B.E., Gupta A., Grinnell B.W., Molitoris B.A. (2009). Soluble thrombomodulin protects ischemic kidneys. J. Am. Soc. Nephrol..

[35] Tietjen G.T., Hosgood S.A., DiRito J., Cui J., Deep D., Song E., Kraehling J.R., Piotrowski-Daspit A.S., Kirkiles-Smith N.C., Al-Lamki R. (2017). Nanoparticle targeting to the endothelium during normothermic machine perfusion of human kidneys. Sci. Transl. Med..

[36] Basile D.P., Friedrich J.L., Spahic J., Knipe N., Mang H., Leonard E.C., Changizi-Ashtiyani S., Bacallao R.L., Molitoris B.A., Sutton T.A. (2011). Impaired endothelial proliferation and mesenchymal transition contribute to vascular rarefaction following acute kidney injury. Am. J. Physiol. Ren. Physiol..

[37] Kwon O., Hong S.M., Ramesh G. (2009). Diminished NO generation by injured endothelium and loss of macula densa nNOS may contribute to sustained acute kidney injury after ischemia-reperfusion. Am. J. Physiol. Ren. Physiol..

[38] Kwon O., Hong S.-M., Sutton T.A., Temm C.J. (2008). Preservation of peritubular capillary endothelial integrity and increasing pericytes may be critical to recovery from postischemic acute kidney injury. Am. J. Physiol. Ren. Physiol..

[39] Steegh F.M., Gelens M.A., Nieman F.H., van Hooff J.P., Cleutjens J.P., van Suylen R.J., Daemen M.J., van Heurn E.L., Christiaans M.H., Peutz-Kootstra C.J. (2011). Early loss of peritubular capillaries after kidney transplantation. J. Am. Soc. Nephrol..

[40] Wicherska-Pawłowska K., Wróbel T., Rybka J. (2021). Toll-Like Receptors (TLRs), NOD-Like Receptors (NLRs), and RIG-I-Like Receptors (RLRs) in Innate Immunity. TLRs, NLRs, and RLRs Ligands as Immunotherapeutic Agents for Hematopoietic Diseases. Int. J. Mol. Sci..

[41] Li D., Wu M. (2021). Pattern recognition receptors in health and diseases. Signal Transduct. Target. Ther..

[42] Zhao H., Perez J.S., Lu K., George A.J., Ma D. (2014). Role of Toll-like receptor-4 in renal graft ischemia-reperfusion injury. Am. J. Physiol. Ren. Physiol..

[43] Wu H., Chen G., Wyburn K.R., Yin J., Bertolino P., Eris J.M., Alexander S.I., Sharland A.F., Chadban S.J. (2007). TLR4 activation mediates kidney ischemia/reperfusion injury. J. Clin. Investig..

[44] Kawasaki T., Kawai T. (2014). Toll-like receptor signaling pathways. Front. Immunol..

[45] Rusai K., Sollinger D., Baumann M., Wagner B., Strobl M., Schmaderer C., Roos M., Kirschning C., Heemann U., Lutz J. (2010). Toll-like receptors 2 and 4 in renal ischemia/reperfusion injury. Pediatr. Nephrol..

[46] Danobeitia J.S., Djamali A., Fernandez L.A. (2014). The role of complement in the pathogenesis of renal ischemia-reperfusion injury and fibrosis. Fibrogenesis Tissue Repair.

[47] Danobeitia J.S., Ziemelis M., Ma X., Zitur L.J., Zens T., Chlebeck P.J., Van Amersfoort E.S., Fernandez L.A. (2017). Complement inhibition attenuates acute kidney injury after ischemia-reperfusion and limits progression to renal fibrosis in mice. PLoS ONE.

[48] Howard M.C., Nauser C.L., Farrar C.A., Sacks S.H. (2021). Complement in ischaemia-reperfusion injury and transplantation. Semin. Immunopathol..

[49] Pefanis A., Ierino F.L., Murphy J.M., Cowan P.J. (2019). Regulated necrosis in kidney ischemia-reperfusion injury. Kidney Int..

[50] Knijff L.W.D., van Kooten C., Ploeg R.J. (2022). The Effect of Hypothermic Machine Perfusion to Ameliorate Ischemia-Reperfusion Injury in Donor Organs. Front. Immunol..

[51] Huber T.B., Edelstein C.L., Hartleben B., Inoki K., Jiang M., Koya D., Kume S., Lieberthal W., Pallet N., Quiroga A. (2012). Emerging role of autophagy in kidney function, diseases and aging. Autophagy.

[52] Schroppel B., Legendre C. (2014). Delayed kidney graft function: From mechanism to translation. Kidney Int..

[53] Rubinsztein D.C., Codogno P., Levine B. (2012). Autophagy modulation as a potential therapeutic target for diverse diseases. Nat. Rev. Drug Discov..

[54] Mariño G., Niso-Santano M., Baehrecke E.H., Kroemer G. (2014). Self-consumption: The interplay of autophagy and apoptosis. Nat. Rev. Mol. Cell Biol..

[55] Chen Q., Kang J., Fu C. (2018). The independence of and associations among apoptosis, autophagy, and necrosis. Signal Transduct. Target. Ther..

[56] Decuypere J.P., Pirenne J., Jochmans I. (2014). Autophagy in renal ischemia-reperfusion injury: Friend or foe?. Am. J. Transplant..

[57] Decuypere J.P., Hutchinson S., Monbaliu D., Martinet W., Pirenne J., Jochmans I. (2020). Autophagy Dynamics and Modulation in a Rat Model of Renal Ischemia-Reperfusion Injury. Int. J. Mol. Sci..

[58] Humphreys B.D., Czerniak S., DiRocco D.P., Hasnain W., Cheema R., Bonventre J.V. (2011). Repair of injured proximal tubule does not involve specialized progenitors. Proc. Natl. Acad. Sci. USA.

[59] Humphreys B.D., Valerius M.T., Kobayashi A., Mugford J.W., Soeung S., Duffield J.S., McMahon A.P., Bonventre J.V. (2008). Intrinsic Epithelial Cells Repair the Kidney after Injury. Cell Stem Cell.

[60] Stamellou E., Leuchtle K., Moeller M.J. (2021). Regenerating tubular epithelial cells of the kidney. Nephrol. Dial. Transplant..

[61] Little M.H., Humphreys B.D. (2022). Regrow or Repair: An Update on Potential Regenerative Therapies for the Kidney. J. Am. Soc. Nephrol..

[62] Kumar S. (2018). Cellular and molecular pathways of renal repair after acute kidney injury. Kidney Int..

[63] Buse M., Moeller M.J., Stamellou E. (2022). What We Have Learned so far From Single Cell Sequencing in Acute Kidney Injury. Front. Physiol..

[64] Gerhardt L.M.S., Koppitch K., van Gestel J., Guo J., Cho S., Wu H., Kirita Y., Humphreys B.D., McMahon A.P. (2023). Lineage Tracing and Single-Nucleus Multiomics Reveal Novel Features of Adaptive and Maladaptive Repair after Acute Kidney Injury. J. Am. Soc. Nephrol..

[65] Kirita Y., Wu H., Uchimura K., Wilson P.C., Humphreys B.D. (2020). Cell profiling of mouse acute kidney injury reveals conserved cellular responses to injury. Proc. Natl. Acad. Sci. USA.

[66] Gerhardt L.M.S., Liu J., Koppitch K., Cippa P.E., McMahon A.P. (2021). Single-nuclear transcriptomics reveals diversity of proximal tubule cell states in a dynamic response to acute kidney injury. Proc. Natl. Acad. Sci. USA.

[67] Naved B.A., Bonventre J.V., Hubbell J.A., Hukriede N.A., Humphreys B.D., Kesselman C., Valerius M.T., McMahon A.P., Shankland S.J., Wertheim J.A. (2022). Kidney repair and regeneration: Perspectives of the NIDDK (Re)Building a Kidney consortium. Kidney Int..

[68] Gerhardt L.M.S., McMahon A.P. (2021). Multi-omic approaches to acute kidney injury and repair. Curr. Opin. Biomed. Eng..

[69] Gerhardt L.M.S., McMahon A.P. (2022). Identifying Common Molecular Mechanisms in Experimental and Human Acute Kidney Injury. Semin. Nephrol..

[70] Situmorang G.R., Sheerin N.S. (2019). Ischaemia reperfusion injury: Mechanisms of progression to chronic graft dysfunction. Pediatr. Nephrol..

[71] Perico N., Cattaneo D., Sayegh M.H., Remuzzi G. (2004). Delayed graft function in kidney transplantation. Lancet.

[72] Singh S.K., Kim S.J. (2016). Epidemiology of Kidney Discard from Expanded Criteria Donors Undergoing Donation after Circulatory Death. J. Am. Soc. Nephrol..

[73] Summers D.M., Johnson R.J., Hudson A., Collett D., Watson C.J., Bradley J.A. (2013). Effect of donor age and cold storage time on outcome in recipients of kidneys donated after circulatory death in the UK: A cohort study. Lancet.

[74] Summers D.M., Johnson R.J., Allen J., Fuggle S.V., Collett D., Watson C.J., Bradley J.A. (2010). Analysis of factors that affect outcome after transplantation of kidneys donated after cardiac death in the UK: A cohort study. Lancet.

[75] Urbanellis P., Mazilescu L., Kollmann D., Linares-Cervantes I., Kaths J.M., Ganesh S., Oquendo F., Sharma M., Goto T., Noguchi Y. (2021). Prolonged warm ischemia time leads to severe renal dysfunction of donation-after-cardiac death kidney grafts. Sci. Rep..

[76] Gill J., Rose C., Lesage J., Joffres Y., Gill J., O’Connor K. (2017). Use and Outcomes of Kidneys from Donation after Circulatory Death Donors in the United States. J. Am. Soc. Nephrol..

[77] Summers D.M., Watson C.J., Pettigrew G.J., Johnson R.J., Collett D., Neuberger J.M., Bradley J.A. (2015). Kidney donation after circulatory death (DCD): State of the art. Kidney Int..

[78] Boyarsky B.J., Jackson K.R., Kernodle A.B., Sakran J.V., Garonzik-Wang J.M., Segev D.L., Ottmann S.E. (2020). Estimating the potential pool of uncontrolled DCD donors in the United States. Am. J. Transplant..

[79] Sanchez-Fructuoso A.I., Perez-Flores I., Del Rio F., Blazquez J., Calvo N., Moreno de la Higuera M.A., Gomez A., Alonso-Lera S., Soria A., Gonzalez M. (2019). Uncontrolled donation after circulatory death: A cohort study of data from a long-standing deceased-donor kidney transplantation program. Am. J. Transplant..

[80] Snoeijs M.G., Winkens B., Heemskerk M.B., Hoitsma A.J., Christiaans M.H., Buurman W.A., van Heurn L.W. (2010). Kidney transplantation from donors after cardiac death: A 25-year experience. Transplantation.

[81] Rijkse E., Ceuppens S., Qi H., Ijzermans J.N., Hesselink D.A., Minnee R.C. (2021). Implementation of donation after circulatory death kidney transplantation can safely enlarge the donor pool: A systematic review and meta-analysis. Int. J. Surg..

[82] Brook N.R., White S.A., Waller J.R., Veitch P.S., Nicholson M.L. (2003). Non-heart beating donor kidneys with delayed graft function have superior graft survival compared with conventional heart-beating donor kidneys that develop delayed graft function. Am. J. Transplant..

[83] de Kok M.J., McGuinness D., Shiels P.G., de Vries D.K., Nolthenius J.B.T., Wijermars L.G., Rabelink T.J., Verschuren L., Stevenson K.S., Kingsmore D.B. (2019). The Neglectable Impact of Delayed Graft Function on Long-term Graft Survival in Kidneys Donated After Circulatory Death Associates with Superior Organ Resilience. Ann. Surg..

[84] Schwarz P., Custódio G., Rheinheimer J., Crispim D., Leitão C.B., Rech T.H. (2018). Brain Death-Induced Inflammatory Activity is Similar to Sepsis-Induced Cytokine Release. Cell Transplant..

[85] Helanterä I., Ibrahim H.N., Lempinen M., Finne P. (2020). Donor Age, Cold Ischemia Time, and Delayed Graft Function. Clin. J. Am. Soc. Nephrol..

[86] Resch T., Cardini B., Oberhuber R., Weissenbacher A., Dumfarth J., Krapf C., Boesmueller C., Oefner D., Grimm M., Schneeberger S. (2020). Transplanting Marginal Organs in the Era of Modern Machine Perfusion and Advanced Organ Monitoring. Front. Immunol..

[87] Jing L., Yao L., Zhao M., Peng L.P., Liu M. (2018). Organ preservation: From the past to the future. Acta Pharmacol. Sin..

[88] Collins G.M., Bravo-Shugarman M., Terasaki P.I. (1969). Kidney preservation for transportation. Initial perfusion and 30 hours’ ice storage. Lancet.

[89] Tingle S.J., Figueiredo R.S., Moir J.A., Goodfellow M., Talbot D., Wilson C.H. (2019). Machine perfusion preservation versus static cold storage for deceased donor kidney transplantation. Cochrane Database Syst. Rev..

[90] Jochmans I., Akhtar M.Z., Nasralla D., Kocabayoglu P., Boffa C., Kaisar M., Brat A., O’Callaghan J., Pengel L.H., Knight S. (2016). Past, Present, and Future of Dynamic Kidney and Liver Preservation and Resuscitation. Am. J. Transplant..

[91] Chatauret N., Coudroy R., Delpech P.O., Vandebrouck C., Hosni S., Scepi M., Hauet T. (2014). Mechanistic analysis of nonoxygenated hypothermic machine perfusion’s protection on warm ischemic kidney uncovers greater eNOS phosphorylation and vasodilation. Am. J. Transplant..

[92] Nordling S., Brännström J., Carlsson F., Lu B., Salvaris E., Wanders A., Buijs J., Estrada S., Tolmachev V., Cowan P.J. (2018). Enhanced protection of the renal vascular endothelium improves early outcome in kidney transplantation: Preclinical investigations in pig and mouse. Sci. Rep..

[93] Vaziri N., Thuillier R., Favreau F.D., Eugene M., Milin S., Chatauret N.P., Hauet T., Barrou B. (2011). Analysis of machine perfusion benefits in kidney grafts: A preclinical study. J. Transl. Med..

[94] Debout A., Foucher Y., Trébern-Launay K., Legendre C., Kreis H., Mourad G., Garrigue V., Morelon E., Buron F., Rostaing L. (2015). Each additional hour of cold ischemia time significantly increases the risk of graft failure and mortality following renal transplantation. Kidney Int..

[95] Jochmans I., Brat A., Davies L., Hofker H.S., van de Leemkolk F.E.M., Leuvenink H.G.D., Knight S.R., Pirenne J., Ploeg R.J. (2020). Oxygenated versus standard cold perfusion preservation in kidney transplantation (COMPARE): A randomised, double-blind, paired, phase 3 trial. Lancet.

[96] Schlegel A., Kron P., Graf R., Clavien P.A., Dutkowski P. (2014). Hypothermic Oxygenated Perfusion (HOPE) downregulates the immune response in a rat model of liver transplantation. Ann. Surg..

[97] Nasralla D., Coussios C.C., Mergental H., Akhtar M.Z., Butler A.J., Ceresa C.D.L., Chiocchia V., Dutton S.J., García-Valdecasas J.C., Heaton N. (2018). A randomized trial of normothermic preservation in liver transplantation. Nature.

[98] Gilmour J., Griffiths C., Pither T., Scott W.E., Fisher A.J. (2020). Normothermic machine perfusion of donor-lungs ex-vivo: Promoting clinical adoption. Curr. Opin. Organ Transplant..

[99] Hosgood S.A., Saeb-Parsy K., Hamed M.O., Nicholson M.L. (2016). Successful Transplantation of Human Kidneys Deemed Untransplantable but Resuscitated by Ex Vivo Normothermic Machine Perfusion. Am. J. Transplant..

[100] Hosgood S.A., Thompson E., Moore T., Wilson C.H., Nicholson M.L. (2018). Normothermic machine perfusion for the assessment and transplantation of declined human kidneys from donation after circulatory death donors. Br. J. Surg..

[101] Mazilescu L.I., Urbanellis P., Kim S.J., Goto T., Noguchi Y., Konvalinka A., Reichman T.W., Sayed B.A., Mucsi I., Lee J.Y. (2022). Normothermic Ex Vivo Kidney Perfusion for Human Kidney Transplantation: First North American Results. Transplantation.

[102] Rijkse E., de Jonge J., Kimenai H., Hoogduijn M.J., de Bruin R.W.F., van den Hoogen M.W.F., Ijzermans J.N.M., Minnee R.C. (2021). Safety and feasibility of 2 h of normothermic machine perfusion of donor kidneys in the Eurotransplant Senior Program. BJS Open.

[103] Hosgood S.A., Barlow A.D., Hunter J.P., Nicholson M.L. (2015). Ex vivo normothermic perfusion for quality assessment of marginal donor kidney transplants. Br. J. Surg..

[104] Weissenbacher A., Lo Faro L., Boubriak O., Soares M.F., Roberts I.S., Hunter J.P., Voyce D., Mikov N., Cook A., Ploeg R.J. (2019). Twenty-four-hour normothermic perfusion of discarded human kidneys with urine recirculation. Am. J. Transplant..

[105] Weissenbacher A., Messner F., Gasteiger S., Soleiman A., Öfner D., Schneeberger S. (2022). Forty-eight hours of normothermic kidney preservation applying urine recirculation. Artif. Organs.

[106] Mazilescu L.I., Urbanellis P., Kaths M.J., Ganesh S., Goto T., Noguchi Y., John R., Konvalinka A., Mucsi I., Ghanekar A. (2021). Prolonged Normothermic Ex Vivo Kidney Perfusion Is Superior to Cold Nonoxygenated and Oxygenated Machine Perfusion for the Preservation of DCD Porcine Kidney Grafts. Transplant. Direct.

[107] Guo Z., Luo T., Mo R., Zhao Q., He X. (2022). Ischemia-free organ transplantation—A review. Curr. Opin. Organ Transplant..

[108] He X., Chen G., Zhu Z., Zhang Z., Yuan X., Han M., Zhao Q., Zheng Y., Tang Y., Huang S. (2019). The First Case of Ischemia-Free Kidney Transplantation in Humans. Front. Med..

[109] Hosgood S.A., Elliott T.R., Jordan N.P., Nicholson M.L. (2022). The Effects of Free Heme on Functional and Molecular Changes During Ex Vivo Normothermic Machine Perfusion of Human Kidneys. Front. Immunol..

[110] Vercaemst L. (2008). Hemolysis in cardiac surgery patients undergoing cardiopulmonary bypass: A review in search of a treatment algorithm. J. Extra Corpor. Technol..

[111] Chiabrando D., Mercurio S., Tolosano E. (2014). Heme and erythropoieis: More than a structural role. Haematologica.

[112] Zakkar M., Guida G., Suleiman M.S., Angelini G.D. (2015). Cardiopulmonary bypass and oxidative stress. Oxid. Med. Cell Longev..

[113] Adams T.D., Patel M., Hosgood S.A., Nicholson M.L. (2017). Lowering Perfusate Temperature From 37 °C to 32 °C Diminishes Function in a Porcine Model of Ex Vivo Kidney Perfusion. Transplant. Direct.

[114] Hoyer D.P., Gallinat A., Swoboda S., Wohlschläger J., Rauen U., Paul A., Minor T. (2014). Subnormothermic machine perfusion for preservation of porcine kidneys in a donation after circulatory death model. Transpl. Int..

[115] Bhattacharjee R.N., Ruthirakanthan A., Sun Q., Richard-Mohamed M., Luke S., Jiang L., Aquil S., Sharma H., Tun-Abraham M.E., Alharbi B. (2019). Subnormothermic Oxygenated Perfusion Optimally Preserves Donor Kidneys Ex Vivo. Kidney Int. Rep..

[116] Salahudeen A.K. (2004). Cold ischemic injury of transplanted kidneys: New insights from experimental studies. Am. J. Physiol. Ren. Physiol..

[117] Sammut I.A., Burton K., Balogun E., Sarathchandra P., Brooks K.J., Bates T.E., Green C.J. (2000). Time-dependent impairment of mitochondrial function after storage and transplantation of rabbit kidneys. Transplantation.

[118] Schopp I., Reissberg E., Lüer B., Efferz P., Minor T. (2015). Controlled Rewarming after Hypothermia: Adding a New Principle to Renal Preservation. Clin. Transl. Sci..

[119] Zulpaite R., Miknevicius P., Leber B., Strupas K., Stiegler P., Schemmer P. (2021). Ex-vivo Kidney Machine Perfusion: Therapeutic Potential. Front. Med..

[120] Smith R.A.J., Hartley R.C., Cochemé H.M., Murphy M.P. (2012). Mitochondrial pharmacology. Trends Pharmacol. Sci..

[121] Granata S., Votrico V., Spadaccino F., Catalano V., Netti G.S., Ranieri E., Stallone G., Zaza G. (2022). Oxidative Stress and Ischemia/Reperfusion Injury in Kidney Transplantation: Focus on Ferroptosis, Mitophagy and New Antioxidants. Antioxidants.

[122] Kezic A., Spasojevic I., Lezaic V., Bajcetic M. (2016). Mitochondria-Targeted Antioxidants: Future Perspectives in Kidney Ischemia Reperfusion Injury. Oxid. Med. Cell. Longev..

[123] Hamed M., Logan A., Gruszczyk A.V., Beach T.E., James A.M., Dare A.J., Barlow A., Martin J., Georgakopoulos N., Gane A.M. (2021). Mitochondria-targeted antioxidant MitoQ ameliorates ischaemia–reperfusion injury in kidney transplantation models. Br. J. Surg..

[124] Wiegman C.H., Michaeloudes C., Haji G., Narang P., Clarke C.J., Russell K.E., Bao W., Pavlidis S., Barnes P.J., Kanerva J. (2015). Oxidative stress-induced mitochondrial dysfunction drives inflammation and airway smooth muscle remodeling in patients with chronic obstructive pulmonary disease. J. Allergy Clin. Immunol..

[125] Dashdorj A., Jyothi K.R., Lim S., Jo A., Nguyen M.N., Ha J., Yoon K.S., Kim H.J., Park J.H., Murphy M.P. (2013). Mitochondria-targeted antioxidant MitoQ ameliorates experimental mouse colitis by suppressing NLRP3 inflammasome-mediated inflammatory cytokines. BMC Med..

[126] Escribano-Lopez I., Diaz-Morales N., Rovira-Llopis S., de Marañon A.M., Orden S., Alvarez A., Bañuls C., Rocha M., Murphy M.P., Hernandez-Mijares A. (2016). The mitochondria-targeted antioxidant MitoQ modulates oxidative stress, inflammation and leukocyte-endothelium interactions in leukocytes isolated from type 2 diabetic patients. Redox Biol..

[127] Birk A.V., Liu S., Soong Y., Mills W., Singh P., Warren J.D., Seshan S.V., Pardee J.D., Szeto H.H. (2013). The Mitochondrial-Targeted Compound SS-31 Re-Energizes Ischemic Mitochondria by Interacting with Cardiolipin. J. Am. Soc. Nephrol..

[128] Zhao K., Zhao G.-M., Wu D., Soong Y., Birk A.V., Schiller P.W., Szeto H.H. (2004). Cell-permeable Peptide Antioxidants Targeted to Inner Mitochondrial Membrane inhibit Mitochondrial Swelling, Oxidative Cell Death, and Reperfusion Injury. J. Biol. Chem..

[129] Szeto H.H., Liu S., Soong Y., Seshan S.V., Cohen-Gould L., Manichev V., Feldman L.C., Gustafsson T. (2017). Mitochondria Protection after Acute Ischemia Prevents Prolonged Upregulation of IL-1β and IL-18 and Arrests CKD. J. Am. Soc. Nephrol..

[130] Saad A., Herrmann S.M.S., Eirin A., Ferguson C.M., Glockner J.F., Bjarnason H., McKusick M.A., Misra S., Lerman L.O., Textor S.C. (2017). Phase 2a Clinical Trial of Mitochondrial Protection (Elamipretide) During Stent Revascularization in Patients With Atherosclerotic Renal Artery Stenosis. Circ. Cardiovasc. Interv..

[131] Chen Y., Fan H., Wang S., Tang G., Zhai C., Shen L. (2021). Ferroptosis: A Novel Therapeutic Target for Ischemia-Reperfusion Injury. Front. Cell Dev. Biol..

[132] Friedmann Angeli J.P., Schneider M., Proneth B., Tyurina Y.Y., Tyurin V.A., Hammond V.J., Herbach N., Aichler M., Walch A., Eggenhofer E. (2014). Inactivation of the ferroptosis regulator Gpx4 triggers acute renal failure in mice. Nat. Cell Biol..

[133] Ni L., Yuan C., Wu X. (2022). Targeting ferroptosis in acute kidney injury. Cell Death Dis..

[134] van den Berg T.A.J., van den Heuvel M.C., Wiersema-Buist J., Adelmeijer J., Nieuwenhuijs-Moeke G.J., Lisman T., Bakker S.J.L., van Goor H., Annema-de Jong J.H., Bakker S.J.L. (2021). Aggravation of fibrin deposition and microthrombus formation within the graft during kidney transplantation. Sci. Rep..

[135] DiRito J.R., Hosgood S.A., Reschke M., Albert C., Bracaglia L.G., Ferdinand J.R., Stewart B.J., Edwards C.M., Vaish A.G., Thiru S. (2021). Lysis of cold-storage-induced microvascular obstructions for ex vivo revitalization of marginal human kidneys. Am. J. Transplant..

[136] Nghiem D.D., Olson P.R., Sureshkumar K.K. (2009). Role of Pulsatile Perfusion With Tissue Plasminogen Activator in Deceased Donor Kidneys With Extensive Glomerular Thrombosis. Transplant. Proc..

[137] Woodside K.J., Goldfarb D.A., Rabets J.C., Sanchez E.Q., Lebovitz D.J., Schulak J.A., Fung J.J., Eghtesad B. (2015). Enhancing kidney function with thrombolytic therapy following donation after cardiac death: A multicenter quasi-blinded prospective randomized trial. Clin. Transplant..

[138] Sedigh A., Nordling S., Carlsson F., Larsson E., Norlin B., Lübenow N., Lennmyr F., Tufveson G., Magnusson P.U., Lorant T. (2019). Perfusion of Porcine Kidneys With Macromolecular Heparin Reduces Early Ischemia Reperfusion Injury. Transplantation.

[139] Hamaoui K., Gowers S., Boutelle M., Cook T.H., Hanna G., Darzi A., Smith R., Dorling A., Papalois V. (2016). Organ Pretreatment With Cytotopic Endothelial Localizing Peptides to Ameliorate Microvascular Thrombosis and Perfusion Deficits in Ex Vivo Renal Hemoreperfusion Models. Transplantation.

[140] Olausson M., Antony D., Travnikova G., Johansson M., Nayakawde N.B., Banerjee D., Søfteland J.M., Premaratne G.U. (2022). Novel Ex-Vivo Thrombolytic Reconditioning of Kidneys Retrieved 4 to 5 Hours After Circulatory Death. Transplantation.

[141] Sedigh A., Lundgren T., Lindnér P., Nordström J., Magnusson P., Jönsson J., Carlsson F., Ploeg R., Lorant T. (2023). Heparin Conjugate Pretreatment of Kidneys From Deceased Donors Before Transplantation: Results From the First-in-human Randomized Phase I Trial. Transplant. Direct.

[142] De Beule J., Keppens D., Korf H., Jochmans I. (2022). Differential Cytokine Levels during Normothermic Kidney Perfusion with Whole Blood- or Red Blood Cell-Based Perfusates-Results of a Scoping Review and Experimental Study. J. Clin. Med..

[143] Hosgood S.A., Moore T., Kleverlaan T., Adams T., Nicholson M.L. (2017). Haemoadsorption reduces the inflammatory response and improves blood flow during ex vivo renal perfusion in an experimental model. J. Transl. Med..

[144] Ferdinand J.R., Hosgood S.A., Moore T., Ferro A., Ward C.J., Castro-Dopico T., Nicholson M.L., Clatworthy M.R. (2021). Cytokine absorption during human kidney perfusion reduces delayed graft function-associated inflammatory gene signature. Am. J. Transplant..

[145] Kielstein J.T., Zarbock A. (2022). Is This the Beginning of the End of Cytokine Adsorption?. Crit. Care Med..

[146] Liu X., Murphy M.P., Xing W., Wu H., Zhang R., Sun H. (2018). Mitochondria-targeted antioxidant MitoQ reduced renal damage caused by ischemia-reperfusion injury in rodent kidneys: Longitudinal observations of T(2) -weighted imaging and dynamic contrast-enhanced MRI. Magn. Reson. Med..

[147] Chen Q., Nan Y., Yang Y., Xiao Z., Liu M., Huang J., Xiang Y., Long X., Zhao T., Wang X. (2023). Nanodrugs alleviate acute kidney injury: Manipulate RONS at kidney. Bioact. Mater..

[148] Feng S., Qu Y., Chu B., Chen X., Yang Z., Li P., Wang P., He Q., He Y., Lin T. (2022). Novel gold-platinum nanoparticles serve as broad-spectrum antioxidants for attenuating ischemia reperfusion injury of the kidney. Kidney Int..

[149] Shin C.S., Han J.U., Kim J.L., Schenarts P.J., Traber L.D., Hawkins H., Traber D.L. (1997). Heparin attenuated neutrophil infiltration but did not affect renal injury induced by ischemia reperfusion. Yonsei Med. J..

[150] Tuuminen R., Jouppila A., Salvail D., Laurent C.E., Benoit M.C., Syrjälä S., Helin H., Lemström K., Lassila R. (2017). Dual antiplatelet and anticoagulant APAC prevents experimental ischemia-reperfusion-induced acute kidney injury. Clin. Exp. Nephrol..

[151] Zhou T., Chen J.L., Song W., Wang F., Zhang M.J., Ni P.H., Geng J.G. (2002). Effect of N-desulfated heparin on hepatic/renal ischemia reperfusion injury in rats. World J. Gastroenterol..

[152] Diuwe P., Domagala P., Durlik M., Trzebicki J., Chmura A., Kwiatkowski A. (2017). The effect of the use of a TNF-alpha inhibitor in hypothermic machine perfusion on kidney function after transplantation. Contemp. Clin. Trials.

[153] Choi D.E., Jeong J.Y., Lim B.J., Na K.R., Shin Y.T., Lee K.W. (2009). Pretreatment with the tumor nerosis factor-alpha blocker etanercept attenuated ischemia-reperfusion renal injury. Transplant. Proc..

[154] Stone J.P., Ball A.L., Critchley W.R., Major T., Edge R.J., Amin K., Clancy M.J., Fildes J.E. (2016). Ex Vivo Normothermic Perfusion Induces Donor-Derived Leukocyte Mobilization and Removal Prior to Renal Transplantation. Kidney Int. Rep..

[155] Kassimatis T., Greenlaw R., Hunter J.P., Douiri A., Flach C., Rebollo-Mesa I., Nichols L.L., Qasem A., Danzi G., Olsburgh J. (2021). Ex vivo delivery of Mirococept: A dose-finding study in pig kidney after showing a low dose is insufficient to reduce delayed graft function in human kidney. Am. J. Transplant..

[156] Danobeitia J.S., Zens T.J., Chlebeck P.J., Zitur L.J., Reyes J.A., Eerhart M.J., Coonen J., Capuano S., D’Alessandro A.M., Torrealba J.R. (2020). Targeted donor complement blockade after brain death prevents delayed graft function in a nonhuman primate model of kidney transplantation. Am. J. Transplant..

[157] Eerhart M.J., Reyes J.A., Blanton C.L., Danobeitia J.S., Chlebeck P.J., Zitur L.J., Springer M., Polyak E., Coonen J., Capuano S. (2022). Complement Blockade in Recipients Prevents Delayed Graft Function and Delays Antibody-mediated Rejection in a Nonhuman Primate Model of Kidney Transplantation. Transplantation.

[158] Huang E., Vo A., Choi J., Ammerman N., Lim K., Sethi S., Kim I., Kumar S., Najjar R., Peng A. (2020). Three-Year Outcomes of a Randomized, Double-Blind, Placebo-Controlled Study Assessing Safety and Efficacy of C1 Esterase Inhibitor for Prevention of Delayed Graft Function in Deceased Donor Kidney Transplant Recipients. Clin. J. Am. Soc. Nephrol..

[159] Jordan S.C., Choi J., Aubert O., Haas M., Loupy A., Huang E., Peng A., Kim I., Louie S., Ammerman N. (2018). A phase I/II, double-blind, placebo-controlled study assessing safety and efficacy of C1 esterase inhibitor for prevention of delayed graft function in deceased donor kidney transplant recipients. Am. J. Transplant..

[160] Schröppel B., Akalin E., Baweja M., Bloom R.D., Florman S., Goldstein M., Haydel B., Hricik D.E., Kulkarni S., Levine M. (2020). Peritransplant eculizumab does not prevent delayed graft function in deceased donor kidney transplant recipients: Results of two randomized controlled pilot trials. Am. J. Transplant..

[161] Bartels-Stringer M., Verpalen J.T., Wetzels J.F., Russel F.G., Kramers C. (2007). Iron chelation or anti-oxidants prevent renal cell damage in the rewarming phase after normoxic, but not hypoxic cold incubation. Cryobiology.

[162] Gonzalez-Fajardo J.A., Fernandez L., Alvarez T., Vaquero C. (1995). Preservation of cortical microcirculation after kidney ischemia-reperfusion: Value of an iron chelator. Ann. Vasc. Surg..

[163] Paller M.S., Hedlund B.E. (1988). Role of iron in postischemic renal injury in the rat. Kidney Int..

[164] de Vries B., Walter S.J., von Bonsdorff L., Wolfs T.G.A.M., van Heurn L.W.E., Parkkinen J., Buurman W.A. (2004). Reduction of circulating redox-active iron by apotransferrin protects against renal ischemia-reperfusion injury1. Transplantation.

[165] Wang Y., Quan F., Cao Q., Lin Y., Yue C., Bi R., Cui X., Yang H., Yang Y., Birnbaumer L. (2021). Quercetin alleviates acute kidney injury by inhibiting ferroptosis. J. Adv. Res..

[166] Linkermann A., Skouta R., Himmerkus N., Mulay S.R., Dewitz C., De Zen F., Prokai A., Zuchtriegel G., Krombach F., Welz P.S. (2014). Synchronized renal tubular cell death involves ferroptosis. Proc. Natl. Acad. Sci. USA.

[167] Ozturk H., Cetinkaya A., Duzcu S.E., Tekce B.K., Ozturk H. (2018). Carvacrol attenuates histopathogic and functional impairments induced by bilateral renal ischemia/reperfusion in rats. Biomed. Pharmacother..

[168] Liu W., Chen B., Wang Y., Meng C., Huang H., Huang X.R., Qin J., Mulay S.R., Anders H.J., Qiu A. (2018). RGMb protects against acute kidney injury by inhibiting tubular cell necroptosis via an MLKL-dependent mechanism. Proc. Natl. Acad. Sci. USA.

[169] Shen B., Mei M., Pu Y., Zhang H., Liu H., Tang M., Pan Q., He Y., Wu X., Zhao H. (2019). Necrostatin-1 Attenuates Renal Ischemia and Reperfusion Injury via Meditation of HIF-1α/mir-26a/TRPC6/PARP1 Signaling. Mol. Ther. Nucleic Acids.

[170] Ashour H., Hashem H.A., Khowailed A.A., Rashed L.A., Hassan R.M., Soliman A.S. (2022). Necrostatin-1 mitigates renal ischaemia-reperfusion injury—Time dependent—Via aborting the interacting protein kinase (RIPK-1)-induced inflammatory immune response. Clin. Exp. Pharmacol. Physiol..

[171] Martens S., Jeong M., Tonnus W., Feldmann F., Hofmans S., Goossens V., Takahashi N., Bräsen J.H., Lee E.W., Van der Veken P. (2017). Sorafenib tosylate inhibits directly necrosome complex formation and protects in mouse models of inflammation and tissue injury. Cell Death Dis..

[172] Pool M.B.F., Vos J., Eijken M., van Pel M., Reinders M.E.J., Ploeg R.J., Hoogduijn M.J., Jespersen B., Leuvenink H.G.D., Moers C. (2020). Treating Ischemically Damaged Porcine Kidneys with Human Bone Marrow- and Adipose Tissue-Derived Mesenchymal Stromal Cells During Ex Vivo Normothermic Machine Perfusion. Stem Cells Dev..

[173] Pool M., Eertman T., Sierra Parraga J., t Hart N., Roemeling-van Rhijn M., Eijken M., Jespersen B., Reinders M., Hoogduijn M., Ploeg R. (2019). Infusing Mesenchymal Stromal Cells into Porcine Kidneys during Normothermic Machine Perfusion: Intact MSCs Can Be Traced and Localised to Glomeruli. Int. J. Mol. Sci..

[174] Lohmann S., Eijken M., Møldrup U., Møller B.K., Hunter J., Moers C., Leuvenink H., Ploeg R.J., Clahsen-van Groningen M.C., Hoogduijn M. (2021). Ex Vivo Administration of Mesenchymal Stromal Cells in Kidney Grafts Against Ischemia-reperfusion Injury-Effective Delivery Without Kidney Function Improvement Posttransplant. Transplantation.

[175] Lohmann S., Pool M.B.F., Rozenberg K.M., Keller A.K., Moers C., Møldrup U., Møller B.K., Lignell S.J.M., Krag S., Sierra-Parraga J.M. (2021). Mesenchymal stromal cell treatment of donor kidneys during ex vivo normothermic machine perfusion: A porcine renal autotransplantation study. Am. J. Transplant..

[176] Arcolino F.O., Hosgood S., Akalay S., Jordan N., Herman J., Elliott T., Veys K., Vermeire K., Sprangers B., Nicholson M. (2022). De novo SIX2 activation in human kidneys treated with neonatal kidney stem/progenitor cells. Am. J. Transplant..

[177] Thompson E.R., Bates L., Ibrahim I.K., Sewpaul A., Stenberg B., McNeill A., Figueiredo R., Girdlestone T., Wilkins G.C., Wang L. (2021). Novel delivery of cellular therapy to reduce ischemia reperfusion injury in kidney transplantation. Am. J. Transplant..

[178] Brasile L., Henry N., Orlando G., Stubenitsky B. (2019). Potentiating Renal Regeneration Using Mesenchymal Stem Cells. Transplantation.

[179] Erpicum P., Rowart P., Poma L., Krzesinski J.-M., Detry O., Jouret F. (2017). Administration of mesenchymal stromal cells before renal ischemia/reperfusion attenuates kidney injury and may modulate renal lipid metabolism in rats. Sci. Rep..

[180] Thompson E.R., Sewpaul A., Figuereido R., Bates L., Tingle S.J., Ferdinand J.R., Situmorang G.R., Ladak S.S., Connelly C.M., Hosgood S.A. (2022). MicroRNA antagonist therapy during normothermic machine perfusion of donor kidneys. Am. J. Transplant..

[181] Zheng X., Feng B., Chen G., Zhang X., Li M., Sun H., Liu W., Vladau C., Liu R., Jevnikar A.M. (2006). Preventing renal ischemia-reperfusion injury using small interfering RNA by targeting complement 3 gene. Am. J. Transplant..

[182] Lobb I., Davison M., Carter D., Liu W., Haig A., Gunaratnam L., Sener A. (2015). Hydrogen Sulfide Treatment Mitigates Renal Allograft Ischemia-Reperfusion Injury during Cold Storage and Improves Early Transplant Kidney Function and Survival Following Allogeneic Renal Transplantation. J. Urol.

[183] Lobb I., Jiang J., Lian D., Liu W., Haig A., Saha M.N., Torregrossa R., Wood M.E., Whiteman M., Sener A. (2017). Hydrogen Sulfide Protects Renal Grafts Against Prolonged Cold Ischemia-Reperfusion Injury via Specific Mitochondrial Actions. Am. J. Transplant..

[184] Bagul A., Hosgood S.A., Kaushik M., Nicholson M.L. (2008). Carbon monoxide protects against ischemia-reperfusion injury in an experimental model of controlled nonheartbeating donor kidney. Transplantation.

[185] Nakao A., Faleo G., Shimizu H., Nakahira K., Kohmoto J., Sugimoto R., Choi A.M., McCurry K.R., Takahashi T., Murase N. (2008). Ex vivo carbon monoxide prevents cytochrome P450 degradation and ischemia/reperfusion injury of kidney grafts. Kidney Int..

[186] Hunter J.P., Hosgood S.A., Patel M., Rose R., Read K., Nicholson M.L. (2012). Effects of hydrogen sulphide in an experimental model of renal ischaemia-reperfusion injury. Br. J. Surg..

[187] Hosgood S.A., Bagul A., Kaushik M., Rimoldi J., Gadepalli R.S., Nicholson M.L. (2008). Application of nitric oxide and carbon monoxide in a model of renal preservation. B.r J. Surg..

[188] Faleo G., Neto J.S., Kohmoto J., Tomiyama K., Shimizu H., Takahashi T., Wang Y., Sugimoto R., Choi A.M., Stolz D.B. (2008). Carbon monoxide ameliorates renal cold ischemia-reperfusion injury with an upregulation of vascular endothelial growth factor by activation of hypoxia-inducible factor. Transplantation.

[189] Hameed A.M., Lu D.B., Burns H., Byrne N., Chew Y.V., Julovi S., Ghimire K., Zanjani N.T., P’Ng C.H., Meijles D. (2020). Pharmacologic targeting of renal ischemia-reperfusion injury using a normothermic machine perfusion platform. Sci. Rep..

[190] Xu M., Wang X., Banan B., Chirumbole D.L., Garcia-Aroz S., Balakrishnan A., Nayak D.K., Zhang Z., Jia J., Upadhya G.A. (2018). Anti-CD47 monoclonal antibody therapy reduces ischemia-reperfusion injury of renal allografts in a porcine model of donation after cardiac death. Am. J. Transplant..

[191] Wang X., Xu M., Jia J., Zhang Z., Gaut J.P., Upadhya G.A., Manning P.T., Lin Y., Chapman W.C. (2018). CD47 blockade reduces ischemia/reperfusion injury in donation after cardiac death rat kidney transplantation. Am. J. Transplant..

[192] Lin Y., Manning P.T., Jia J., Gaut J.P., Xiao Z., Capoccia B.J., Chen C.C., Hiebsch R.R., Upadhya G., Mohanakumar T. (2014). CD47 blockade reduces ischemia-reperfusion injury and improves outcomes in a rat kidney transplant model. Transplantation.

[193] Stewart B.J., Ferdinand J.R., Young M.D., Mitchell T.J., Loudon K.W., Riding A.M., Richoz N., Frazer G.L., Staniforth J.U.L., Vieira Braga F.A. (2019). Spatiotemporal immune zonation of the human kidney. Science.

[194] Turner J.-E., Becker M., Mittrücker H.-W., Panzer U. (2018). Tissue-Resident Lymphocytes in the Kidney. J. Am. Soc. Nephrol..

[195] Boardman D.A., Jacob J., Smyth L.A., Lombardi G., Lechler R.I. (2016). What Is Direct Allorecognition?. Curr. Transplant. Rep..

[196] Marino J., Babiker-Mohamed M.H., Crosby-Bertorini P., Paster J.T., LeGuern C., Germana S., Abdi R., Uehara M., Kim J.I., Markmann J.F. (2016). Donor exosomes rather than passenger leukocytes initiate alloreactive T cell responses after transplantation. Sci. Immunol..

[197] Zhou W., Farrar C.A., Abe K., Pratt J.R., Marsh J.E., Wang Y., Stahl G.L., Sacks S.H. (2000). Predominant role for C5b-9 in renal ischemia/reperfusion injury. J. Clin. Investig..

[198] de Vries B., Matthijsen R.A., Wolfs T.G.A.M., van Bijnen A.A.J.H.M., Heeringa P., Buurman W.A. (2003). Inhibition of complement factor C5 protects against renal ischemia-reperfusion injury: Inhibition of late apoptosis and inflammation1. Transplantation.

[199] Calcat I.C.S., Sanz-Nogues C., O’Brien T. (2021). When Origin Matters: Properties of Mesenchymal Stromal Cells From Different Sources for Clinical Translation in Kidney Disease. Front. Med..

[200] Sindberg G.M., Lindborg B.A., Wang Q., Clarkson C., Graham M., Donahue R., Hering B.J., Verfaillie C.M., Bansal-Pakala P., O’Brien T.D. (2014). Comparisons of phenotype and immunomodulatory capacity among rhesus bone-marrow-derived mesenchymal stem/stromal cells, multipotent adult progenitor cells, and dermal fibroblasts. J. Med. Primatol..

[201] Khan R.S., Newsome P.N. (2019). A Comparison of Phenotypic and Functional Properties of Mesenchymal Stromal Cells and Multipotent Adult Progenitor Cells. Front. Immunol..

[202] Arcolino F.O., Zia S., Held K., Papadimitriou E., Theunis K., Bussolati B., Raaijmakers A., Allegaert K., Voet T., Deprest J. (2016). Urine of Preterm Neonates as a Novel Source of Kidney Progenitor Cells. J. Am. Soc. Nephrol..

[203] Little M.H., Lawlor K.T. (2020). Recreating, expanding and using nephron progenitor populations. Nat. Rev. Nephrol..

[204] Little M.H., Kairath P. (2017). Does Renal Repair Recapitulate Kidney Development?. J. Am. Soc. Nephrol..

[205] Eggenhofer E., Benseler V., Kroemer A., Popp F., Geissler E., Schlitt H., Baan C., Dahlke M., Hoogduijn M. (2012). Mesenchymal stem cells are short-lived and do not migrate beyond the lungs after intravenous infusion. Front. Immunol..

[206] de Witte S.F.H., Luk F., Sierra Parraga J.M., Gargesha M., Merino A., Korevaar S.S., Shankar A.S., O’Flynn L., Elliman S.J., Roy D. (2018). Immunomodulation By Therapeutic Mesenchymal Stromal Cells (MSC) Is Triggered Through Phagocytosis of MSC By Monocytic Cells. Stem Cells.

[207] Scarfe L., Taylor A., Sharkey J., Harwood R., Barrow M., Comenge J., Beeken L., Astley C., Santeramo I., Hutchinson C. (2018). Non-invasive imaging reveals conditions that impact distribution and persistence of cells after in vivo administration. Stem Cell Res. Ther..

[208] Galleu A., Riffo-Vasquez Y., Trento C., Lomas C., Dolcetti L., Cheung T.S., von Bonin M., Barbieri L., Halai K., Ward S. (2017). Apoptosis in mesenchymal stromal cells induces in vivo recipient-mediated immunomodulation. Sci. Transl. Med..

[209] Pang S.H.M., D’Rozario J., Mendonca S., Bhuvan T., Payne N.L., Zheng D., Hisana A., Wallis G., Barugahare A., Powell D. (2021). Mesenchymal stromal cell apoptosis is required for their therapeutic function. Nat. Commun..

[210] Grange C., Bussolati B. (2022). Extracellular vesicles in kidney disease. Nat. Rev. Nephrol..

[211] Corrêa R.R., Juncosa E.M., Masereeuw R., Lindoso R.S. (2021). Extracellular Vesicles as a Therapeutic Tool for Kidney Disease: Current Advances and Perspectives. Int. J. Mol. Sci..

[212] Gregorini M., Corradetti V., Pattonieri E.F., Rocca C., Milanesi S., Peloso A., Canevari S., De Cecco L., Dugo M., Avanzini M.A. (2017). Perfusion of isolated rat kidney with Mesenchymal Stromal Cells/Extracellular Vesicles prevents ischaemic injury. J. Cell Mol. Med..

[213] Rampino T., Gregorini M., Germinario G., Pattonieri E.F., Erasmi F., Grignano M.A., Bruno S., Alomari E., Bettati S., Asti A. (2022). Extracellular Vesicles Derived from Mesenchymal Stromal Cells Delivered during Hypothermic Oxygenated Machine Perfusion Repair Ischemic/Reperfusion Damage of Kidneys from Extended Criteria Donors. Biology.

[214] Ruan Y., Wang L., Zhao Y., Yao Y., Chen S., Li J., Guo H., Ming C., Chen S., Gong F. (2014). Carbon monoxide potently prevents ischemia-induced high-mobility group box 1 translocation and release and protects against lethal renal ischemia-reperfusion injury. Kidney Int..

[215] Nakao A., Neto J.S., Kanno S., Stolz D.B., Kimizuka K., Liu F., Bach F.H., Billiar T.R., Choi A.M., Otterbein L.E. (2005). Protection against ischemia/reperfusion injury in cardiac and renal transplantation with carbon monoxide, biliverdin and both. Am. J. Transplant..

[216] Lobb I., Mok A., Lan Z., Liu W., Garcia B., Sener A. (2012). Supplemental hydrogen sulphide protects transplant kidney function and prolongs recipient survival after prolonged cold ischaemia-reperfusion injury by mitigating renal graft apoptosis and inflammation. BJU Int..

[217] Moody B.F., Calvert J.W. (2011). Emergent role of gasotransmitters in ischemia-reperfusion injury. Med. Gas Res..

[218] Hauet T., Thuillier R. (2017). Protecting the Mitochondria Against Ischemia Reperfusion: A Gassy Solution?. Am. J. Transplant..

[219] Damase T.R., Sukhovershin R., Boada C., Taraballi F., Pettigrew R.I., Cooke J.P. (2021). The Limitless Future of RNA Therapeutics. Front. Bioeng. Biotechnol..

[220] Bondue T., van den Heuvel L., Levtchenko E., Brock R. (2023). The potential of RNA-based therapy for kidney diseases. Pediatr. Nephrol..

[221] Lam J.K., Chow M.Y., Zhang Y., Leung S.W. (2015). siRNA Versus miRNA as Therapeutics for Gene Silencing. Mol. Ther. Nucleic Acids.

[222] Yang B., Hosgood S.A., Nicholson M.L. (2011). Naked small interfering RNA of caspase-3 in preservation solution and autologous blood perfusate protects isolated ischemic porcine kidneys. Transplantation.

[223] Yang C., Jia Y., Zhao T., Xue Y., Zhao Z., Zhang J., Wang J., Wang X., Qiu Y., Lin M. (2013). Naked caspase 3 small interfering RNA is effective in cold preservation but not in autotransplantation of porcine kidneys. J. Surg. Res..

[224] Zheng X., Zhang X., Sun H., Feng B., Li M., Chen G., Vladau C., Chen D., Suzuki M., Min L. (2006). Protection of renal ischemia injury using combination gene silencing of complement 3 and caspase 3 genes. Transplantation.

[225] Anselmo A.C., Mitragotri S. (2019). Nanoparticles in the clinic: An update. Bioeng. Transl. Med..

[226] Waheed S., Li Z., Zhang F., Chiarini A., Armato U., Wu J. (2022). Engineering nano-drug biointerface to overcome biological barriers toward precision drug delivery. J. Nanobiotechnol..

[227] Blanco E., Shen H., Ferrari M. (2015). Principles of nanoparticle design for overcoming biological barriers to drug delivery. Nat. Biotechnol..

[228] Mitchell M.J., Billingsley M.M., Haley R.M., Wechsler M.E., Peppas N.A., Langer R. (2021). Engineering precision nanoparticles for drug delivery. Nat. Rev. Drug Discov..

[229] Gao L., Zhuang J., Nie L., Zhang J., Zhang Y., Gu N., Wang T., Feng J., Yang D., Perrett S. (2007). Intrinsic peroxidase-like activity of ferromagnetic nanoparticles. Nat. Nanotechnol..

[230] Zhang D.-Y., Liu H., Younis M.R., Lei S., Yang C., Lin J., Qu J., Huang P. (2021). Ultrasmall platinum nanozymes as broad-spectrum antioxidants for theranostic application in acute kidney injury. Chem. Eng. J..

[231] Isenberg J.S., Roberts D.D. (2019). The role of CD47 in pathogenesis and treatment of renal ischemia reperfusion injury. Pediatr. Nephrol..

[232] Hosszu A., Fekete A., Szabo A.J. (2020). Sex differences in renal ischemia-reperfusion injury. Am. J. Physiol. Ren. Physiol..

[233] Dixon E.E., Wu H., Muto Y., Wilson P.C., Humphreys B.D. (2022). Spatially Resolved Transcriptomic Analysis of Acute Kidney Injury in a Female Murine Model. J. Am. Soc. Nephrol..

